# Cancer Incidence in Women After Medically Assisted Reproduction

**DOI:** 10.1001/jamanetworkopen.2026.1332

**Published:** 2026-03-10

**Authors:** Claire Melissa Vajdic, Adrian Raymond Walker, Antoinette C. Anazodo, Neville F. Hacker, Michael Chapman, Signe Opdahl, Louisa Jorm, Robert J. Norman, Catharyn Stern, Ursula M. Sansom-Daly, Georgina Mary Chambers, Christos Venetis

**Affiliations:** 1 Kirby Institute, University of New South Wales, Sydney, New South Wales, Australia; 2 Centre for Big Data Research in Health, University of New South Wales, Sydney, New South Wales, Australia; 3 Kids Cancer Centre, Sydney Children’s Hospital, Randwick, New South Wales, Australia; 4 Women’s Health, Paediatrics & Child Health, School of Clinical Medicine, University of New South Wales, Sydney, New South Wales, Australia; 5 IVF Australia Southern Sydney, Sydney, New South Wales, Australia; 6 St George Hospital, Sydney, New South Wales, Australia; 7 Department of Public Health and Nursing, Faculty of Medicine and Health Sciences, Norwegian University of Science and Technology, Trondheim, Norway; 8 Robinson Research Institute, Faculty of Health and Medical Sciences, University of Adelaide, Adelaide, South Australia, Australia; 9 Melbourne IVF, Melbourne, Victoria, Australia; 10 Department of Obstetrics, Gynaecology and Newborn Health, University of Melbourne, Melbourne, Victoria, Australia; 11 Behavioural Sciences Unit, Discipline of Paediatrics and Child Health, School of Clinical Medicine, Faculty of Medicine and Health, UNSW, Randwick, New South Wales, Australia; 12 Sydney Youth Cancer Service, Nelune Comprehensive Cancer Centre, Prince of Wales Hospital, Randwick, New South Wales, Australia; 13 Unit for Human Reproduction, 1st Dept of Ob/Gyn, Medical School, Faculty of Health Sciences, Aristotle University of Thessaloniki, Thessaloniki, Greece

## Abstract

**Importance:**

High-quality evidence on cancer occurrence for women who have used medically assisted reproduction (MAR) is required to guide care.

**Objective:**

To compare cancer incidence in MAR-exposed women with the general population of women.

**Design, Setting, and Participants:**

This is a population-based cohort study of Australian women. MAR treatments, pregnancies, incident cancers, and deaths were ascertained using linkage between population-based administrative health datasets and statutory registries. Women aged 18 to 55 years who used MAR from 1991 to 2018 were identified and analyzed from April to November 2024.

**Exposures:**

Three MAR cohorts were created: assisted reproductive therapy (ART), intrauterine insemination with ovarian stimulation (IUI/OS), and ovulation induction using clomiphene citrate (clomiphene citrate).

**Main Outcomes and Measures:**

Cancer incidence among MAR-exposed women was compared with the age-, jurisdiction-, and calendar year–matched general population. The main outcomes were cancer standardized incidence ratios (SIRs) and rate differences.

**Results:**

A total of 417 984 MAR-exposed women were included, with 274 676 (65.7%) having ever used ART (median [IQR] age, 34 [31-38] years; median [IQR] follow-up time, 9.42 [5.08-15.42] years), 120 739 (28.9%) having ever used IUI/OS (median [IQR] age, 34 [30-38] years; median [IQR] follow-up time, 11.67 [6.25-18.42] years), and 175 510 (42.0%) having ever used clomiphene citrate (median [IQR] age, 32 [28-36] years; median [IQR] follow-up time, 9.42 [5.42-13.58] years). The overall incidence of invasive cancer was comparable with the general population for the ART (SIR, 1.00; 95% CI, 0.98-1.02) and IUI/OS (SIR, 0.99; 95% CI, 0.97-1.02) cohorts and slightly elevated for the clomiphene citrate cohort (SIR, 1.04; 95% CI, 1.00-1.07). For all cohorts, incidence of uterine cancer (SIRs, 1.23-1.83) and in situ and invasive melanoma (SIRs, 1.07-1.15) were elevated, and incidence of cervical cancer (SIRs, 0.52-0.61) and cancer of the trachea, bronchus, and lung (SIRs, 0.62-0.70) were lower. Ovarian cancer incidence was elevated for the ART (SIR, 1.23; 95% CI, 1.10-1.37) and IUI/OS (SIR, 1.18; 95% CI, 1.01-1.37) cohorts. In situ breast cancer incidence was elevated for the ART cohort only (SIR, 1.24; 95% CI, 1.12-1.38). Incidence of invasive breast cancer was not elevated. Rate differences for invasive cancers with elevated incidence were all small (<1 to 6.51 cases per 100 000 person-years).

**Conclusions and Relevance:**

In this cohort study of cancer incidence in women who received MAR, the overall incidence of cancer was comparable with that of the general population. The incidence of certain cancers appeared elevated; however, the excess numbers of these cancers were small, and there was reduced incidence of other cancers. Causation cannot be inferred from this descriptive evidence, but findings may guide women and their health care practitioners.

## Key Points

**Question:**

Is the incidence of cancer in women exposed to medically assisted reproduction (MAR) higher than in the general population of women?

**Findings:**

In this cohort study with 417 984 women exposed to MAR in Australia from 1991 to 2018, the all-cancer incidence was not different to the age-, jurisdiction-, and calendar year–matched general population of women. Incidence for some hormone-sensitive cancers (uterine, ovarian) and cutaneous melanoma were higher, while incidence for cervical cancer and cancer of the trachea, bronchus, and lung were significantly reduced.

**Meaning:**

In this study, certain cancers appeared to be slightly more common in Australian women who have used MAR, and this evidence should enhance awareness, cancer risk behavior modification (reduce adiposity, sun exposure, smoking), and clinical guidelines for follow-up care.

## Teaser

This cohort study evaluates whether women in Australia who have used assisted reproductive therapy, intrauterine insemination with ovarian stimulation, or clomiphene citrate have a higher incidence of cancer than the general population of women.

## Introduction

The number of people receiving medically assisted reproduction (MAR) is increasing in high-income countries. In Australia in 2017, 6.7% of births were conceived by MAR.^1^ MAR treatments include assisted reproductive technologies (ART), such as in-vitro fertilization (IVF) and intracytoplasmic sperm injection (ICSI); intrauterine insemination (IUI); and ovulation induction using fertility medications (eg, clomiphene citrate).

There is longstanding concern that ovarian and other hormone-sensitive cancers are more common in women exposed to MAR.^2,3,4,5^ Potentially carcinogenic MAR exposures include fertility medications and repeated puncture of ovarian follicles during IVF. However, other factors may explain any elevated incidences of these cancers in MAR-exposed women, including reproductive history and use of hormonal contraceptives,^6,7,8^ and causes of female infertility, such as endometriosis^9^ and polycystic ovary syndrome.^10^ Women having MAR after a cancer diagnosis may carry a genetic predisposition and may also be at increased risk of malignant neoplasms from cancer therapies.^11,12^ On the other hand, compared with the general population, women exposed to ART are less likely to smoke^13^ and are more likely to be of high socioeconomic status.^14^ Heightened medical surveillance before, during, and after MAR^15,16,17^ may also affect the age and stage at cancer diagnosis.

Population-based studies of cancer incidence indicate ovarian cancer may be more common, and cervical cancer less common, in women exposed to ART compared with the general population.^3,18,19,20,21,22,23^ Findings for other cancers are mixed.^3,18,19,20,21,22,23^ Robust data on the incidence of all cancers informs women and health care professionals before and after use of MAR. This cohort study compared the incidence of cancer in women exposed to MAR with that of the general population of Australian women over a 28-year period.

## Methods

The study was approved by human research ethics committees (HRECs), including the AIHW HREC, and researchers accessed anonymized data. The HRECs deemed the project met the required guidelines for a waiver of informed consent. The Strengthening the Reporting of Observational Studies in Epidemiology (STROBE) reporting guideline for cohort studies were followed.^24^

### Study Population

While the term *women* is used, this project includes all people assigned female at birth, including gender diverse and nonbinary people. We used linked jurisdictional and national population-based administrative and registry datasets. MAR-exposed cohorts were derived by the Australian Institute of Health and Welfare (AIHW) (eTable 1 in Supplement 1) using probabilistic data linkage. Women aged 18 to 55 years between January 1, 1991, and December 31, 2018, were eligible and identified from the directory of Australians registered to receive Medicare. Medicare is Australia’s public health care insurance scheme, of which all citizens and permanent residents are eligible. This file was linked to the Medicare Benefits Schedule and Pharmaceutical Benefits Scheme datasets to identify women with a reimbursement claim for MAR-related services and/or medications (eTable 2 in Supplement 1). As pharmaceutical claims data were only available from July 1, 2002, the capture period for clomiphene citrate dispensations began then. Deaths were ascertained by linkage with the National Death Index (1991-2019).

**Figure 1.**
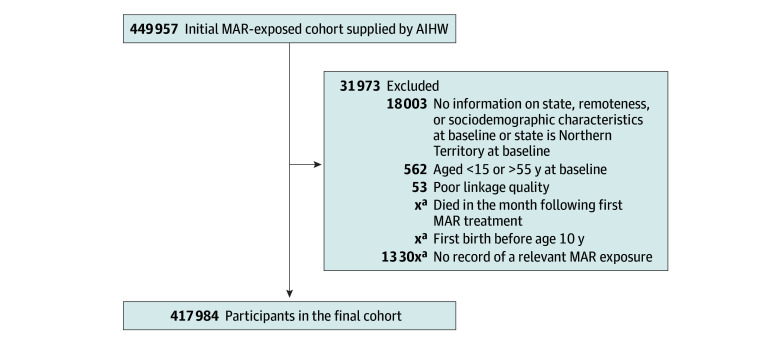
Cohort Derivation Flowchart MAR indicates medically assisted reproduction. ^a^Censored to prevent reidentification from small cell sizes.

The study period varied by Australian jurisdiction in line with dates of coverage of their perinatal data collection (eTable 1 in Supplement 1). The final cohort was formed after exclusion of duplicate, illogical, and missing records as well as the application of exclusion criteria (Figure 1). Women residing in one small Australian jurisdiction (Northern Territory) when they received MAR were excluded as no perinatal or birth data could be provided, and parity is a key predictor of risk for some cancers.

### Exposure Definition

Three MAR exposure cohorts were defined: (1) ART treatments (IVF, ICSI), hereafter referred to as ART; (2) IUI with ovarian stimulation (OS) with follicle-stimulating hormone or ART cancelled before egg retrieval, hereafter referred to as IUI/OS; and (3) ovulation induction using clomiphene citrate, hereafter referred to as clomiphene citrate. Cancelled ART cycles were grouped with IUI with OS because both groups were exposed to ovarian stimulation medicine. As women may be exposed to more than 1 MAR type over time, these cohorts were not mutually exclusive. Women contributed person-time to a cohort from their date of first exposure to a given treatment but were not censored if they later received another MAR treatment.

### Other Variable Definitions

The variables derived at study entry (first MAR treatment) were age, calendar year, jurisdiction, residential remoteness, and area-based indices of socioeconomic status. Time-varying variables included attained age (18-34, 35-39, 40-44, 45-49, 50-54, and ≥55 years), calendar year, parity (nulliparous, parous), and years since first MAR treatment (<1, 1-2, 3-5, >5). Parity or pregnancy history was derived from jurisdictional perinatal and birth registries (eMethods 1 in Supplement 1), noting the perinatal data included pregnancies reaching 20 weeks’ gestation, with some minor variations between jurisdictions (eTable 1 in Supplement 1).

### Outcome Ascertainment

AIHW linked data on MAR-exposed women to the population-based Australian Cancer Database (1982-2019). Cancers are statutory reportable events, and the data are high quality.^25^

Cancers were predefined using *International Classification for Diseases–Oncology *version 3.2 topography and morphology codes (eTable 3 in Supplement 1). All registered cancers were malignant, except for melanoma and breast, for which in situ cancers were also ascertained and were notifiable from January 1, 2002, and January 1, 2004, respectively. Cutaneous basal cell carcinoma and squamous cell carcinoma are not notifiable in all Australian jurisdictions and were not examined.

### Statistical Analysis

Women were followed up from the date of their first relevant MAR exposure until either December 31, 2019, or death, whichever occurred first. Standardized incidence ratios (SIRs), measures of relative risk compared with the general population, were standardized by age group, jurisdiction, and calendar year and calculated for each cancer and MAR cohort. National cancer rates for women, stratified by the standardization variables, were generated by the AIHW from the Australian Cancer Database. SIR 95% CIs were calculated using exact Poisson count tests. For select cancers (breast, uterine, ovarian, melanoma, colorectal, and thyroid), we conducted a post hoc exploratory analysis in which SIRs were stratified by attained age, calendar year, parity, number of MAR treatments, and years since first treatment. Parity was available for the study period for the MAR cohorts only, before and after treatment. Rate differences for each cancer were calculated per 100 000 person-years, from the derived SIR and the expected number of cancers.

The original analysis plan is available online.^26^ Analyses were performed April 2024 to November 2024 using a combination of SAS version 9.4 (SAS Institute), R version 4.4.2 (R Project for Statistical Computing), and Stata version 19 (StataCorp); the code is online.^27^

## Results

### Cohort Demographics

A total of 417 984 women were identified as exposed to MAR in the study period. The ART (274 676 women [65.7%]), IUI/OS (120 739 women [28.9%]), and clomiphene citrate (175 510 women [42.0%]) cohorts were followed up for a median (IQR) of 9.42 (5.08-15.42), 11.67 (6.25-18.42), and 9.42 (5.42-13.58) years, respectively, and the median (IQR) ages at first MAR were 34 (31-38), 34 (30-38), and 32 (28-36) years, respectively (Table). Across all cohorts, women had a median of 2 treatments of the same type during follow-up, and half were first exposed after 2010. Approximately 79% of women in the ART and IUI/OS cohorts were nulliparous at first MAR, compared with 69% in the clomiphene citrate cohort. Distribution by state of residence largely mirrored that for the general population. Approximately 83% of women receiving ART or IUI/OS lived in a major city, compared with 78% of women receiving clomiphene citrate. For all cohorts, most women lived in areas of lowest socioeconomic disadvantage. The mortality rate was lowest for the clomiphene citrate cohort.

**Table.** Cohort Demographic Characteristics by MAR Cohort

Characteristic	Participants, by MAR cohort No. (%)		
	ART (n = 274 676)	IUI/OS (n = 120 739)	Clomiphene citrate (n = 175 510)
Age at study entry, median (IQR), y	34 (31-38)	34 (30-38)	32 (28-36)
Year of study entry			
1991-1994	16 479 (6.0)	10 263 (8.5)	NA
1995-1999	24 351 (8.9)	16 275 (13.5)	NA
2000-2004	34 510 (12.6)	18 843 (15.6)	32 328 (18.4)
2005-2009	60 425 (22.0)	25 185 (20.9)	52 179 (29.7)
2010-2014	71 593 (26.1)	26 816 (22.2)	56 284 (32.1)
2015-2018	67 318 (24.5)	23 357 (19.3)	34 719 (19.8)
Australian state or territory of residence			
Australian Capital Territory	5116 (1.9)	3044 (2.5)	2867 (1.6)
New South Wales	96 817 (35.2)	38 767 (32.1)	58 560 (33.4)
Queensland	48 666 (17.7)	31 339 (26.0)	38 917 (22.2)
South Australia	19 161 (7.0)	6398 (5.3)	10 557 (6.0)
Tasmania	4998 (1.8)	2425 (2.0)	3012 (1.7)
Victoria	75 279 (27.4)	26 245 (21.7)	51 235 (29.2)
Western Australia	24 639 (9.0)	12 521 (10.4)	10 362 (5.9)
Residential remoteness			
Major city	226 494 (82.5)	100 288 (83.1)	136 663 (77.9)
Inner regional	32 290 (11.8)	14 385 (11.9)	25 936 (14.8)
Outer regional	13 236 (4.8)	5331 (4.4)	10 841 (6.2)
Remote	1849 (0.7)	533 (0.4)	1503 (0.9)
Very remote	807 (0.3)	202 (0.2)	567 (0.3)
Area-based index of socioeconomic disadvantage quintile			
1 (Most disadvantaged)	33 528 (12.2)	14 611 (12.1)	29 608 (16.9)
2	35 882 (13.1)	15 160 (12.6)	27 293 (15.6)
3	50 857 (18.5)	22 800 (18.9)	35 730 (20.4)
4	60 682 (22.1)	27 716 (23.0)	36 766 (20.9)
5 (Least disadvantaged)	93 727 (34.1)	40 452 (33.5)	46 113 (26.3)
Birth before study entry			
No	219 759 (80.0)	94 508 (78.3)	121 199 (69.1)
Yes	54 917 (20.0)	26 231 (21.7)	54 311 (30.9)
Birth after study entry			
No	120 322 (43.8)	57 000 (47.2)	55 423 (31.6)
Yes	154 354 (56.2)	63 739 (52.8)	120 087 (68.4)
Years of follow-up			
Mean (SD)	11.00 (7.22)	12.68 (7.64)	9.70 (4.73)
Median (IQR)	9.42 (5.08-15.42)	11.67 (6.25-18.42)	9.42 (5.42-13.58)
Total No. of treatments during follow-up^a^			
Mean (SD)	2.96 (2.81)	2.41 (2.42)	2.75 (2.51)
Median (IQR)	2 (1-4)	2 (1-3)	2 (1-3)
Died during follow-up			
No	272 640 (99.3)	119 698 (99.1)	174 756 (99.6)
Yes	2036 (0.7)	1041 (0.9)	754 (0.4)
Record of treatment in study period			
ART	274 676 (100)	78 463 (65.0)	61 356 (42.4)
IUI/OS	78 463 (28.6)	120 739 (100)	37.309 (21.3)
Clomiphene citrate	61 356 (22.3)	37 309 (30.9)	175 510 (100)

Abbreviations: ART, assisted reproductive technology; IUI/OS, intrauterine insemination with ovarian stimulation; MAR, medically assisted reproduction; NA, not applicable.

^a^ One treatment was defined as the presence of at least 1 relevant exposure code in a given 30-day period (starting from the first treatment day).

### SIRs

**Figure 2.**
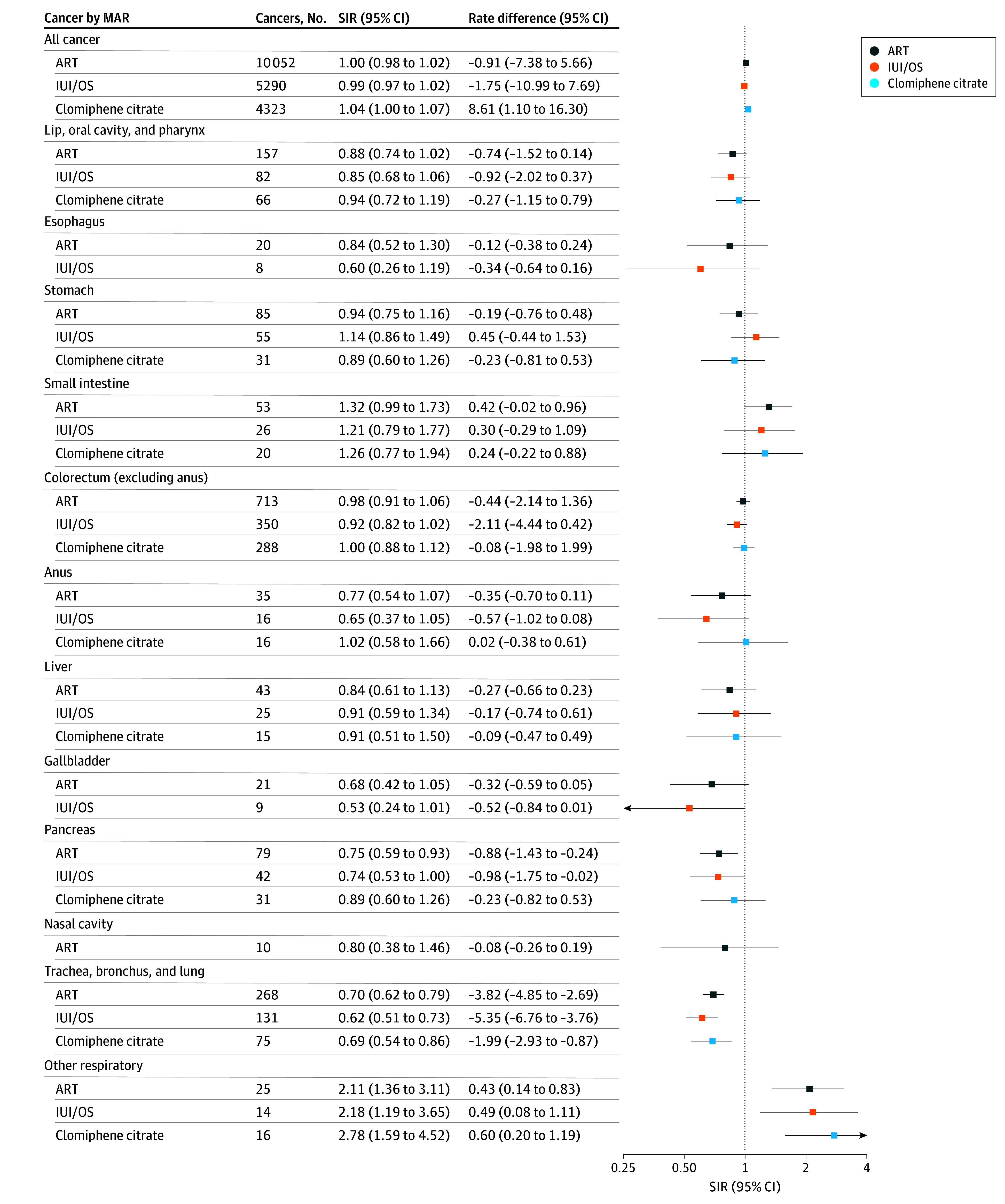
Forest Plot of Cancer Standardized Incidence Ratios (SIRs) and Cancer Rate Differences, Stratified by Medically Assisted Reproduction (MAR) Type for All Cancer, Colorectal Cancer, and Trachea, Bronchus, and Lung Cancer, Among Others ART indicates assisted reproductive therapy; IUI/OS, intrauterine insemination with ovarian stimulation.

**Figure 3.**
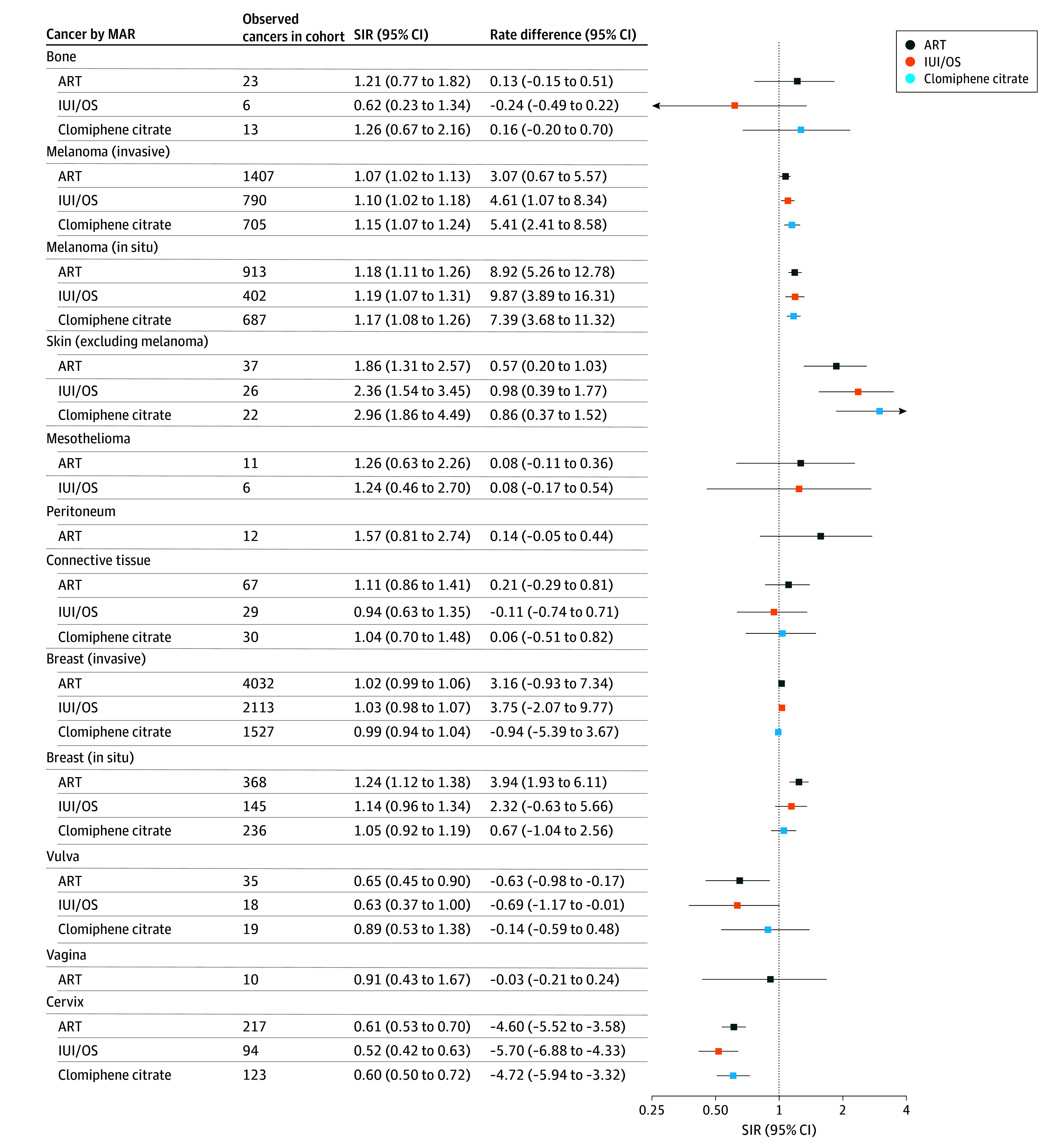
Forest Plot of Cancer Standardized Incidence Ratios (SIRs) and Cancer Rate Differences, Stratified by Medically Assisted Reproduction (MAR) Type for Skin, Breast, and Cervical Cancers, Among Others ART indicates assisted reproductive therapy; IUI/OS, intrauterine insemination with ovarian stimulation.

**Figure 4.**
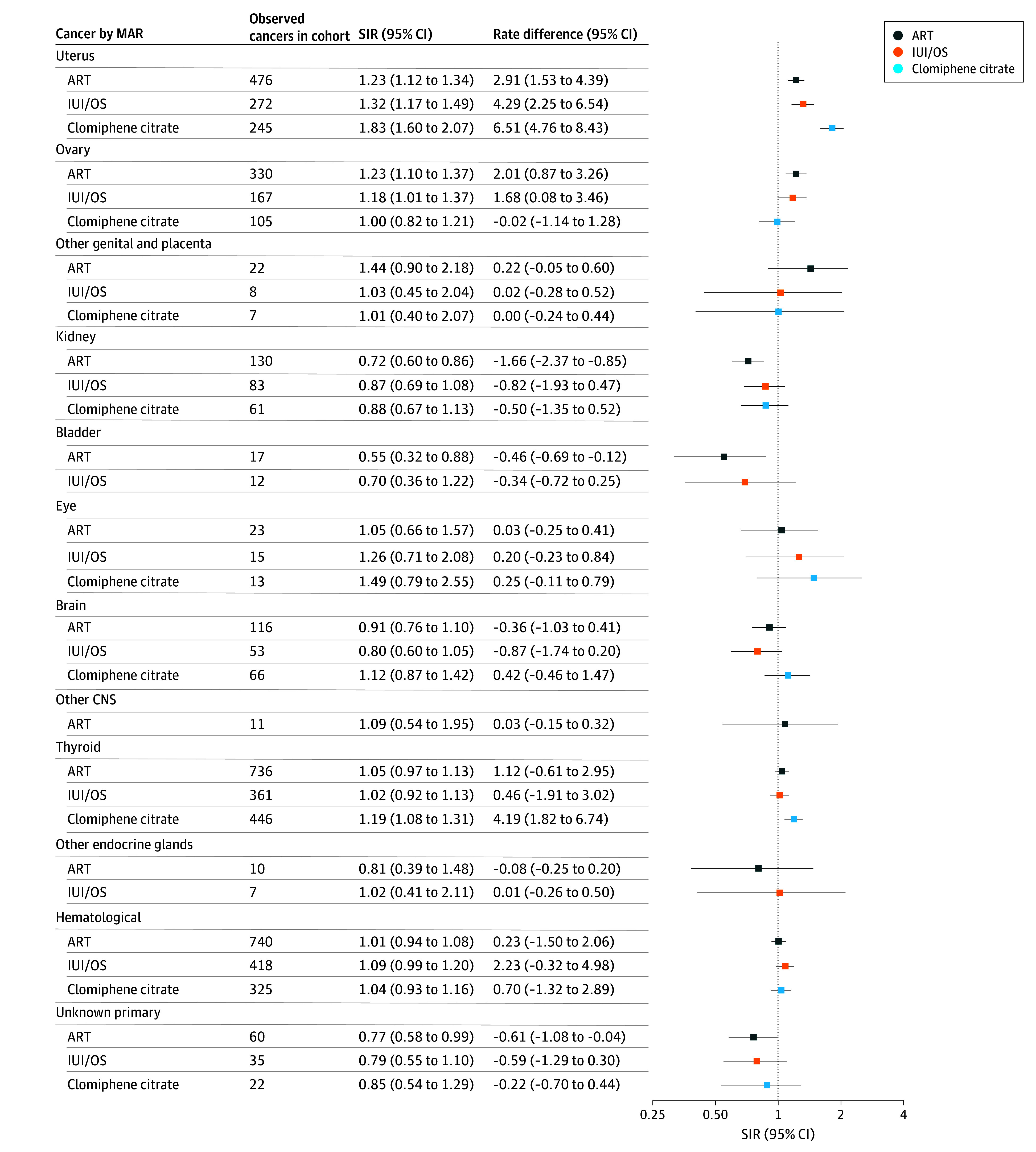
Forest Plot of Cancer Standardized Incidence Ratios (SIRs) and Cancer Rate Differences, Stratified by Medically Assisted Reproduction (MAR) Type for Uterine, Ovarian, and Thyroid Cancers, Among Others ART indicates assisted reproductive therapy; CNS, central nervous system; IUI/OS, intrauterine insemination with ovarian stimulation.

The overall incidence of invasive cancer was comparable with that of the general population for the ART (SIR, 1.00; 95% CI, 0.98-1.02), IUI/OS (SIR, 0.99; 95% CI, 0.97-1.02) and clomiphene citrate (SIR, 1.04; 95% CI, 1.00-1.07) cohorts. Findings for individual cancers were variable (Figures 2, 3, and 4; eTable 4 and eFigures 1-11 in Supplement 1).

#### Hormone-Related Cancers

Invasive breast cancer incidence was similar to the general population for each MAR cohort overall, but it differed in some strata (Figure 3). It was moderately reduced only within 1 year of first treatment for all cohorts and slightly elevated for women of attained age 50 years or older and 6 or more years since first treatment in the ART and IUI/OS cohorts (eFigure 1 in Supplement 1).

In situ breast cancer incidence was elevated for the ART cohort only (SIR, 1.24; 95% CI, 1.12-1.38) (Figure 3). Within this cohort, a similar excess incidence was observed regardless of attained age, parity, and number of cycles (up to 6), but for time since first treatment, incidence was only elevated from 6 years on (eFigure 2 in Supplement 1).

Uterine cancer was more common in women exposed to any type of MAR than the general population, with an SIR of 1.23 (95% CI, 1.12-1.34) for ART, 1.32 (95% CI, 1.17-1.49) for IUI/OS, and 1.83 (95% CI, 1.60-2.07) for clomiphene citrate (Figure 4). For all MAR cohorts, the excess incidence appeared confined to type 1, specifically endometrioid cancer, and was highest the first year after treatment (eFigure 3 in Supplement 1). Incidence was elevated for most groups within the ART and IUI/OS cohorts, with no gradients with respect to attained age or number of treatments. Incidence was strongly elevated for all strata within the clomiphene citrate cohort (SIRs, 1.43-3.83), with one exception, parous women (SIR, 0.88; 95% CI, 0.69-1.11). Within the clomiphene citrate cohort, incidence was highest for women of attained age 18 to 35 years and within 1 year of first treatment.

Compared with the general population, ovarian cancer incidence was elevated for women exposed to ART (SIR, 1.23; 95% CI, 1.10-1.37) and IUI/OS (SIR, 1.18; 95% CI, 1.01-1.37) and appeared restricted to the endometrioid and serous subtypes (Figure 4; eFigure 4 in Supplement 1). For all MAR cohorts, incidence was highest among women with 6 or more treatments and within a year of treatment, moderately increased for nulliparous women (SIRs, 1.48-1.67), and moderately reduced for parous women (SIRs, 0.63-0.80).

Melanoma was slightly more common in all MAR cohorts than in the general population, whether invasive (SIR for ART, 1.07; 95% CI, 1.02-1.13) or in situ (SIR for ART, 1.18; 95% CI, 1.11-1.26) (Figure 3). Incidence was consistently higher for parous women compared with the general population, with no patterns by attained age, number of treatments, or years since first treatment (eFigures 5 and 6 in Supplement 1). The incidence of colorectal cancer was neither elevated nor decreased for the MAR cohorts compared with the general population (Figure 2; eFigure 7 in Supplement 1).

#### Other Cancers

Thyroid cancer was slightly more common in women exposed to clomiphene citrate than the general population (SIR, 1.19; 95% CI, 1.08-1.31) (Figure 4). The excess incidence was only evident for parous women and from 3 to 5 years since first treatment (eFigure 8 in Supplement 1).

All MAR cohorts had a strongly decreased incidence of cervical cancer (SIR for ART, 0.61; 95% CI, 0.53-0.70) and a decreased or similar incidence of other anogenital cancers (eg, SIR for cancer of the vulva for ART, 0.64; 95% CI, 0.45-0.90). All MAR cohorts exhibited a decreased incidence of trachea, bronchus, and lung cancer (SIR for ART, 0.70; 95% CI, 0.62-0.79) (Figure 2).

Pancreatic cancer incidence was moderately decreased for the ART (SIR, 0.75; 95% CI, 0.59-0.93) and IUI/OS (SIR, 0.74; 95% CI, 0.53-1.00) cohorts, and kidney, bladder, and unknown primary cancer incidence was decreased for the ART cohort only (SIR for kidney, 0.72; 95% CI, 0.60-0.86; SIR for bladder, 0.55; 95% CI, 0.32-0.88; and SIR for unknown, 0.77; 95% CI, 0.58-0.99); the SIRs for these cancers were less than unity for all other MAR cohorts.

All MAR cohorts had a moderately elevated incidence of cancer registry–notifiable skin cancer other than melanoma (SIR for ART, 1.86; 95% CI, 1.31-2.57), most commonly mycosis fungoides as well as cancer of the thymus, heart, mediastinum, pleura, and other and ill-defined sites. For all MAR cohorts, there was no excess incidence of any hematological cancer or major hematological cancer subtype. The incidence of acute myeloid leukemia was strongly decreased in all MAR cohorts (SIR for ART, 0.41; 95% CI, 0.28-0.60), and chronic myeloid leukemia incidence was moderately increased in the ART (SIR, 1.39; 95% CI, 1.02-1.85) and IUI/OS (SIR, 1.69; 95% CI, 1.13-2.43) cohorts (eFigure 11 in Supplement 1).

### Rate Differences

The slight excess all-cancer incidence in the clomiphene citrate cohort corresponded to 8.6 (95% CI, 1.1 to 16.3) more cancers per 100 000 person-years (Figures 2, 3, and 4). For invasive cancers with elevated rates, the additional cases of specific cancer types ranged from less than 1 per 100 000 person-years for nonlung respiratory and nonmelanoma skin cancers to 3.1 per 100 000 person-years for melanoma in the ART cohort, less than 1 per 100 000 person-years for nonlung respiratory and nonmelanoma skin cancers to 4.6 per 100 000 person-years for melanoma in the IUI/OS cohort, and less than 1 per 100 000 person-years for nonlung respiratory and nonmelanoma skin cancers to 6.5 per 100 000 person-years for uterine cancer in the clomiphene citrate cohort. For invasive cancers with decreased rates, the corresponding fewer cancer cases per 100 000 person-years were less than 1 for pancreatic, vulvar, bladder, and unknown primary cancers to 4.6 for cervical cancer in the ART cohort, less than 1 for pancreatic and vulvar cancers to 5.7 for cervical cancer in the IUI/OS cohort, and 2.0 for trachea, bronchus, and lung cancer to 4.7 for cervical cancer in the clomiphene citrate cohort.

## Discussion

In this study, Australian women who have used MAR had comparable overall incidence of cancer as the general population. This reassuring headline finding comprises some cancers with excess and others with decreased incidence. Except for uterine cancer and rare skin and respiratory tract cancers, the excess incidence was slight, as were the numbers of excess cancer cases. Although most of the small number of cancers occurring more commonly in women exposed to MAR are known to be associated with exogenous hormones or hormonal changes, causation cannot be inferred from this descriptive evidence, in which the comparator population is the general population of women matched only for age, calendar year, and state of residence. The variation in cancer incidence with pregnancy history, and the absence of consistent gradients in incidence with increasing MAR exposure, indicate explanatory factors other than MAR, including type of infertility, adiposity, skin type, and smoking history, which are unavailable in the dataset. The results may help MAR-treated women and their health care practitioners manage their modifiable cancer risk factors and guide preventive health care and early cancer detection.

Compared with the general population, uterine cancer, melanoma, nonmelanoma skin cancer, and nonlung respiratory cancers were more common for women exposed to any MAR. Extra caution is required for the latter 2 cancer groups because of the small numbers of incident cancers observed. One or more MAR cohorts also had an excess incidence of in situ breast, ovarian, and thyroid cancer as well as chronic myeloid leukemia. Contrastingly, all MAR cohorts had a decreased incidence of cervical and lung cancer as well as acute myeloid leukemia, and some had a decreased incidence of vulvar, pancreatic, kidney, bladder, and unknown primary site cancer.

Importantly, clinical investigations for infertility prior to MAR will lead to the detection of cancers and precancerous lesions, which would theoretically result in the MAR cohort having a lower incidence of cancer than the general population after the start of treatment.^4,28^ On the other hand, after the first MAR treatment, women are under greater medical monitoring than the general population, which may lead to cancers being diagnosed at an earlier age. This includes the frequent use of pelvic imaging to monitor the response to ovarian stimulation, treatment complications, and the health of reproductive organs and fetuses. The excess risk observed within a year of MAR exposure for uterine and ovarian cancer may therefore represent medical surveillance rather than a treatment effect.

Unlike this study, 3 prior population-based studies reported no significant excess incidence of uterine cancer after MAR compared with the general population.^3,18,22^ Like this study, the largest prior study detected an increased incidence for nulliparous women, no variation with respect to the number of treatments, and a predominance of endometrioid tumors.^22^ Elevated uterine cancer incidence for women using fertility treatments is not unexpected given the established positive associations with underlying causes of infertility and anovulation.^9,10,29^ The high increased incidence of uterine cancer in all subsets of women exposed to clomiphene citrate, except for parous women, should necessitate early reporting and investigation of relevant symptoms, but does not resolve the long-standing concern that this medication may increase uterine cancer risk.^30^

Prior evidence from the United States and the Netherlands showed no excess incidence of melanoma in women exposed to MAR compared with the general population.^18,19^ The slight excess incidence of both invasive and in situ melanoma in our study supports heightened awareness for this malignant neoplasm in the Australian MAR population. However, these findings may represent selection bias, specifically women using MAR being more likely to have a fairer complexion compared with the general population. Although there are no such data on Australian women undertaking MAR, the available evidence indicates lower uptake of infertility treatment by Aboriginal and Torres Strait Islander women than non-Indigenous women,^31,32^ and internationally, women seeking medical help for fertility issues are more likely to be White.^33^ In addition to socioeconomic factors related to MAR access, the predominance of Caucasian women may be related to lower MAR awareness among people from non-European countries and possibly cultural and religious barriers to seeking infertility treatment.^34,35^ Detection bias associated with greater health care access is another explanation.

Prior studies of breast cancer incidence compared with the general population show mixed findings, reporting no excess incidence,^3,21^ excess incidence only for in situ breast cancer,^22^ and decreased incidence.^18^ Considering the findings from this study, the balance of evidence is reassuring and does not support varying population breast screening guidance for women after MAR.

An increased incidence of ovarian cancer after ART is consistent with evidence from the UK^22^ but not the US,^18^ or previous estimates from Australia^3^ where incidence was the same as the general population. The largest prior study also detected an increased incidence for nulliparous women, and no gradient with respect to the number of treatments or time since treatment.^22^ The current balance of evidence supports basing MAR-treated women’s health care and awareness of ovarian cancer risk on their pregnancy history and underlying cause of infertility.

Despite excess thyroid cancer incidence in women with endometriosis,^9^ and a large proportion of thyroid cancers being detected incidentally^36^ and detection bias being likely for all MAR treatments, this study found an increased incidence of thyroid cancer only for women who used clomiphene citrate. Like thyroid cancer, hematological cancers have not been examined in previous population-based studies, and the variations in incidence of leukemia subtypes are novel and require confirmation.

The observed decreased incidence of cervical cancer compared with the general population was anticipated based on prior reports^18^ and has been attributed to screening and treatment during fertility investigations. Routine cervical screening must be maintained during follow-up care in accordance with population guidance.

To our knowledge, this is the first study to identify a decreased incidence of several major cancers for women who have used MAR, including lung and pancreatic cancer and, for women exposed to ART, also kidney and bladder cancer. Findings for these strongly smoking-related cancers accord with evidence that women exposed to ART are less likely to have smoked.^13^

### Strengths and Limitations

This study has strengths, including its use of high-quality and objective population-based claims and registry datasets to ascertain MAR exposures and cancer diagnoses, including registerable invasive and in situ tumors. Additionally, the findings are broadly generalizable to countries with similar MAR eligibility criteria.

However, this study also has limitations. It was unable to assess cancer incidence by stage (including borderline cancers), cancer incidence compared with infertile women unexposed to MAR or in relation to health care access, or cause-specific cancer mortality. There is no person-level national data on the indication for fertility treatment, adiposity, breastfeeding, skin type, or smoking, preventing the estimation of incidence stratified by these features. There is currently no means of identifying a population-based cohort of infertile women unexposed to MAR in Australia. We note multiple testing may have increased the risk of chance findings in our stratified analyses, and SIR estimates in many strata had low precision due to small numbers of observed cancers. Additionally, the follow-up duration is relatively short, and most women were younger than 50 years at the end of follow-up.

Treatment trajectories for women who receive MAR are heterogenous. We opted to include all women who received a relevant MAR treatment, regardless of treatment history or treatment trajectory. In doing so, we capture the population-level incidence of cancer in those who received treatment at any point in time. This approach allows us to describe the burden of cancer in these populations but cannot be used to infer risk for individuals.

## Conclusions

In this cohort study of cancer incidence in women who received MAR, Australian women who used MAR had the same overall incidence of cancer as the matched general population. The excess incidence of specific cancers warrants personalized risk management and follow-up care.

## Notes

Supplement 1. **eMethods.** Derivation of Parous and Nulliparous **eTable 1.** Description of Datasets Used to Identify MAR Exposures, Parity, Incident Cancer, and Death **eTable 2.** Claim Codes Used to Identify Each Type of MAR **eTable 3.** International Classification of Diseases for Oncology (ICD-O) Codes Used to Classify Incident Cancers **eTable 4.** SIRs and Rate Differences for All Cancers for Those Exposed to Medically Assisted Reproduction **eFigure 1.** Forest Plot of SIRs and Rate Differences (Per 100 000 Person-Years) for Invasive Breast Cancer by MAR Cohort and Time-Varying Woman and Treatment Characteristics **eFigure 2.** Forest Plot of SIRs and Rate Differences (Per 100 000 Person-Years) for In Situ Breast Cancer by MAR Cohort and Time-Varying Woman and Treatment Characteristics **eFigure 3.** Forest Plot of SIRs and Rate Differences (Per 100 000 Person-Years) for Uterine Cancer by MAR Cohort and Time-Varying Woman and Treatment Characteristics **eFigure 4.** Forest Plot of SIRs and Rate Differences (Per 100 000 Person-Years) for Ovarian Cancer by MAR Cohort and Time-Varying Woman and Treatment Characteristics **eFigure 5.** Forest Plot of SIRs and Rate Differences (Per 100 000 Person-Years) for Invasive Melanoma by MAR Cohort and Time-Varying Woman and Treatment Characteristics **eFigure 6.** Forest Plot of SIRs and Rate Differences (Per 100 000 Person-Years) for In Situ Melanoma by MAR Cohort and Time-Varying Woman and Treatment Characteristics **eFigure 7.** Forest Plot of SIRs and Rate Differences (Per 100 000 Person-Years) for Colorectal Cancer by MAR Cohort and Time-Varying Woman and Treatment Characteristics **eFigure 8.** Forest Plot of SIRs and Rate Differences (Per 100 000 Person-Years) for Thyroid Cancer by MAR Cohort and Time-Varying Woman and Treatment Characteristics **eFigure 9.** Forest Plot of SIRs and Rate Difference (Per 100 000 Person-Years) for Uterine Cancer Morphology Subtypes by MAR Cohort **eFigure 10.** Forest Plot of SIRs and Rate Difference (Per 100 000 Person-Years) for Ovarian Cancer Morphology Subtypes by MAR Cohort **eFigure 11.** Forest Plot of SIRs and Rate Difference (Per 100 000 Person-Years) for Hematological Cancer Subtypes by MAR Cohort

Supplement 2.
Data Sharing Statement
