# Supplementary material for: Cancer Incidence in Women After Medically Assisted Reproduction
Source: JAMA Netw Open. 2026 Mar 10;9(3):e261332. doi: 10.1001/jamanetworkopen.2026.1332 (PMC12976796; doi:10.1001/jamanetworkopen.2026.1332)
Supplement: Supplement 1. — eMethods. Derivation of Parous and Nulliparous eTable 1. Description of Datasets Used to Identify MAR Exposures, Parity, Incident Cancer, and Death eTable 2. Claim Codes Used to Identify Each Type of MAR eTable 3. International Classification of Diseases for Oncology (ICD-O) Codes Used to Classify Incident Cancers eTable 4. SIRs and Rate Differences for All Cancers for Those Exposed to Medically Assisted Reproduction eFigure 1. Forest Plot of SIRs and Rate Differences (Per 100 000 Person-Years) for Invasive Breast Cancer by MAR Cohort and Time-Varying Woman and Treatment Characteristics eFigure 2. Forest Plot of SIRs and Rate Differences (Per 100 000 Person-Years) for In Situ Breast Cancer by MAR Cohort and Time-Varying Woman and Treatment Characteristics eFigure 3. Forest Plot of SIRs and Rate Differences (Per 100 000 Person-Years) for Uterine Cancer by MAR Cohort and Time-Varying Woman and Treatment Characteristics eFigure 4. Forest Plot of SIRs and Rate Differences (Per 100 000 Person-Years) for Ovarian Cancer by MAR Cohort and Time-Varying Woman and Treatment Characteristics eFigure 5. Forest Plot of SIRs and Rate Differences (Per 100 000 Person-Years) for Invasive Melanoma by MAR Cohort and Time-Varying Woman and Treatment Characteristics eFigure 6. Forest Plot of SIRs and Rate Differences (Per 100 000 Person-Years) for In Situ Melanoma by MAR Cohort and Time-Varying Woman and Treatment Characteristics eFigure 7. Forest Plot of SIRs and Rate Differences (Per 100 000 Person-Years) for Colorectal Cancer by MAR Cohort and Time-Varying Woman and Treatment Characteristics eFigure 8. Forest Plot of SIRs and Rate Differences (Per 100 000 Person-Years) for Thyroid Cancer by MAR Cohort and Time-Varying Woman and Treatment Characteristics eFigure 9. Forest Plot of SIRs and Rate Difference (Per 100 000 Person-Years) for Uterine Cancer Morphology Subtypes by MAR Cohort eFigure 10. Forest Plot of SIRs and Rate Difference (Per 100 000 Person-Years) for Ovarian Cancer Morphol [file jamanetwopen-e261332-s001.pdf]

# Supplemental Online Content

Vajdic CM, Walker AR, Anazodo AC, et al. Cancer incidence in women after medically assisted reproduction. *JAMA Netw Open.* 2026;9(3):e261332. doi:10.1001/jamanetworkopen.2026.1332

**eMethods.** Derivation of Parous and Nulliparous

**eTable 1.** Description of Datasets Used to Identify MAR Exposures, Parity, Incident Cancer, and Death

**eTable 2.** Claim Codes Used to Identify Each Type of MAR

**eTable 3.** International Classification of Diseases for Oncology (ICD-O) Codes Used to Classify Incident Cancers

**eTable 4.** SIRs and Rate Differences for All Cancers for Those Exposed to Medically Assisted Reproduction

**eFigure 1.** Forest Plot of SIRs and Rate Differences (Per 100 000 Person-Years) for Invasive Breast Cancer by MAR Cohort and Time-Varying Woman and Treatment Characteristics

**eFigure 2.** Forest Plot of SIRs and Rate Differences (Per 100 000 Person-Years) for In-Situ Breast Cancer by MAR Cohort and Time-Varying Woman and Treatment Characteristics

**eFigure 3.** Forest Plot of SIRs and Rate Differences (Per 100 000 Person-Years) for Uterine Cancer by MAR Cohort and Time-Varying Woman and Treatment Characteristics

**eFigure 4.** Forest Plot of SIRs and Rate Differences (Per 100 000 Person-Years) for Ovarian Cancer by MAR Cohort and Time-Varying Woman and Treatment Characteristics

**eFigure 5.** Forest Plot of SIRs and Rate Differences (Per 100 000 Person-Years) for Invasive Melanoma by MAR Cohort and Time-Varying Woman and Treatment Characteristics

**eFigure 6.** Forest Plot of SIRs and Rate Differences (Per 100 000 Person-Years) for In-Situ Melanoma by MAR Cohort and Time-Varying Woman and Treatment Characteristics

**eFigure 7.** Forest Plot of SIRs and Rate Differences (Per 100 000 Person-Years) for Colorectal Cancer by MAR Cohort and Time-Varying Woman and Treatment Characteristics

**eFigure 8.** Forest Plot of SIRs and Rate Differences (Per 100 000 Person-Years) for Thyroid Cancer by MAR Cohort and Time-Varying Woman and Treatment Characteristics

© 2026 Vajdic CM et al. *JAMA Network Open*.

**eFigure 9.** Forest Plot of SIRs and Rate Difference (Per 100 000 Person-Years) for Uterine Cancer Morphology Subtypes by MAR Cohort

**eFigure 10.** Forest Plot of SIRs and Rate Difference (Per 100 000 Person-Years) for Ovarian Cancer Morphology Subtypes by MAR Cohort

**eFigure 11.** Forest Plot of SIRs and Rate Difference (Per 100 000 Person-Years) for Hematological Cancer Subtypes by MAR Cohort

This supplemental material has been provided by the authors to give readers additional information about their work.

**eMethods 1. Derivation of Parous and Nulliparous**

Parity was derived from a combination of births recorded in the jurisdictional Registries of Births, Deaths, and Marriages and their corresponding Perinatal Data Collections (PDCs). We note it was not possible to ascertain births outside of Australia or births prior to the years of available PDC records, and we also note the potential for data entry errors. To reduce the impact of these data constraints, parity was coded to “parous” (parity = 1) or “nulliparous” (parity = 0) using the following rules:

1. If there were no birth or perinatal records, consider a woman “Nulliparous” for the whole study.
2. If a birth record linked to a PDC record had a recorded parity value indicating the first birth, and there were no birth records before that, consider it the first birth. Code “Nulliparous” before this date and “Parous” after this date. This date could occur either before or after entry to a MAR cohort.
3. If the first recorded birth had a parity linked to the PDC that was greater than 1, then consider all analysis time prior to this birth as “Parous”.

We note missing records mean parity will be misclassified for some women; for example, a missing record for a woman’s only recorded birth would mean she was coded as nulliparous for the whole study. The occurrence of these errors will increase closer to the start date of the jurisdictional PDCs, because births prior to these dates will not be captured in this study and, in the absence of further births to that woman, no information on parity will be recorded.

© 2026 Vajdic CM et al. *JAMA Network Open*.

**eTable 1. Description of Datasets Used to Identify MAR Exposures, Parity, Incident Cancer, and Death**

| Dataset | Timeframe | Original purpose and setting | Use in this study |
|---|---|---|---|
| **National datasets** | | | |
| Medicare Enrolment File (MEF)[a] | 1 Jan 1991 - 31 Dec 2019 | Administrative data collection of all individuals enrolled in Medicare, Australia's national public health insurance program, including names and addresses. Almost all Australians are enrolled soon after birth; immigrants are enrolled when they become eligible. | Used to identify women eligible for cohort entry and derive information related to residential geographic location (state, rurality, and socioeconomic status). |
| Medicare Benefits Schedule (MBS)[b] | 1 Jan 1991 - 31 Dec 2019 | Administrative data collection of all health service claims through the Medicare Benefits Schedule. Data provided by healthcare providers who claim from the scheme. | Used to identify women who received a MAR treatment in the study period. Only MAR-relevant items provided to researchers (See eTable 2). |
| Pharmaceutical Benefits Scheme (PBS)[c] | 1 Jul 2002 - 31 Dec 2019 | Administrative data collection of all drugs dispensed through the Pharmaceutical Benefits Scheme, Australia's national public drug reimbursement program. Data provided by pharmacists who claim from the scheme. | Used to identify women who were dispensed a MAR-relevant drug in the study period, and the type of drug (See eTable 2). |
| National Death Index (NDI)[d] | 1 Jan 1991 - 31 Dec 2019 | Mandated registry of all deaths occurring in Australia. Obtained from the Australian Institute of Health and Welfare. | Used to identify when people died to censor person-time at risk in analysis. |
| Australian Cancer Database (ACD)[e] | 1 Jan 1982 - 31 Dec 2019 | Collection of all notifiable cancers notified to the Australia population-based cancer registries. | Used to identify when cancer was diagnosed, as well as the type of cancer (topography and morphology). |
| **State datasets** | | | |
| Perinatal Data Collections (PDCs) for NSW, ACT, QLD, VIC, WA[f], and TAS | **NSW:** 1 Jan 1994 – 30 Jun 2019<br>**ACT:** 1 Jan 1997 – 1 Dec 2017 | Administrative data collections of all pregnancies, generally beyond 20 weeks gestation. Victoria also includes pregnancies above 400 grams in weight at birth if not over 20 weeks. State collections differ, but include date of birth, status of the birth (live or still), | Used to determine women's history of pregnancy, including previous children and parity of time at study entry, and pregnancy characteristics. |

© 2026 Vajdic CM et al. *JAMA Network Open*.

| Dataset | Timeframe | Original purpose and setting | Use in this study |
|---|---|---|---|
| | **QLD:** 1 Jan 2007 - 31 Dec 2019<br>**VIC:** 1 Jan 1999 - 20 Oct 2022<br>**SA:** 1 Jan 1991 - 31 Dec 2019<br>**WA:** 1 Jan 1991 - 31 Dec 2020<br>**TAS:** 1 Jan 2005 - 31 Dec 2019 | plurality, parity, complications of pregnancy, and other relevant data items. | |
| Registry of Births, Deaths, and Marriages (RBDM) for NSW, ACT, QLD, VIC, WA, and TAS | **NSW:** 1 Jan 1994 – 30 Jun 2019<br>**ACT:** 1 Jan 1997 – 1 Dec 2017<br>**QLD:** 1 Jan 2007 - 31 Dec 2018<br>**VIC:** 1 Jan 1999 - 20 Oct 2022<br>**SA:** 1 Jan 1991 - 31 Dec 2019<br>**WA:** 1 Jan 1991 - 31 Dec 2020<br>**TAS:** 1 Jan 2005 - 31 Dec 2019 | Mandated registers of all births recorded in each State and Territory, including information on mothers and fathers. | Used in combination with the perinatal data collections to determine details of pregnancy history and parity. |

NSW: New South Wales; ACT: Australian Capital Territory; QLD: Queensland; VIC: Victoria; SA: South Australia; WA: Western Australia; TAS: Tasmania.
a Australian National Audit Office (ANAO). Integrity of Medicare Enrolment Data. 2024. Accessed August 18, 2025. https://www.anao.gov.au/work/performance-audit/integrity-medicare-enrolment-data
b Australian Institute of Health and Welfare. Medicare Benefits Schedule data collection. 2024. Accessed August 18 2025. https://www.aihw.gov.au/about-our-data/our-data-collections/medicare-benefits-schedule-mbs
c Australian Institute of Health and Welfare. Pharmaceutical Benefits Scheme data collection. 2024. Accessed August 18, 2025. https://www.aihw.gov.au/about-our-data/our-data-collections/pharmaceutical-benefits-scheme
d Australian Institute of Health and Welfare. National Death Index. 2024. Accessed August 18, 2025. https://www.aihw.gov.au/about-our-data/our-data-collections/national-death-index
e Australian Institute of Health and Welfare. Australian Cancer Database. 2024. Accessed August 18, 2025. https://www.aihw.gov.au/about-our-data/our-data-collections/australian-cancer-database
f Known as the “Western Australia Midwife Notification System”.

© 2026 Vajdic CM et al. *JAMA Network Open*.

## eTable 2. Claim Codes Used to Identify Each Type of MAR

| Type of MAR | MBS codes[a, b] | PBS code[a] |
|---|---|---|
| **Before January 2010** | | |
| Any MAR | Any of the below categories | |
| ART | 13200 (Assisted reproductive technologies superovulated treatment cycle proceeding to oocyte retrieval – initial cycle in a single calendar year) OR<br>13206 (Assisted reproductive technologies treatment cyle using either the natural cycle or oral medication only to induce oocyte growth and development) OR<br>13212 (Oocyte retrieval for the purpose of assisted reproductive technologies) | |
| IUI/OS | 13203 (Ovulation monitoring services for artificial insemination or gonadotrophin, stimulated ovulation induction) | |
| Clomiphene citrate[c] | | 01211R (from 1 July 2002, excluding 1 Jan 2009 to 30 Jun 2009) |
| **After January 2010** | | |
| Any MAR | Any of the below categories | |
| ART | 13200 (Assisted reproductive technologies superovulated treatment cycle proceeding to oocyte retrieval – initial cycle in a single calendar year) OR<br>13201 (Assisted reproductive technologies superovulated treatment cycle proceeding to oocyte retrieval – subsequent cycle in a single calendar year) OR<br>13206 (Assisted reproductive technologies treatment cyle using either the natural cycle or oral medication only to induce oocyte growth and development) OR<br>13212 (Oocyte retrieval for the purpose of assisted reproductive technologies) | |
| IUI/OS | 13202 (Assisted reproductive technologies superovulated treatment cycle that is cancelled before oocyte retrieval involving the use of drugs to induce superovulation) OR<br>13203 (Ovulation monitoring services for artificial insemination or gonadotrophin, stimulated ovulation induction) | |
| Clomiphene citrate[c] | | 01211R (from 1 July 2002, excluding 1 Jan 2012 to 30 Apr 2012) |

MAR: Medically assisted reproduction; MBS: Medicare Benefits Schedule; PBS: Pharmaceutical Benefits Scheme
a Presence or one or more of these codes within a 30-day window in the relevant dataset indicated the receipt of the relevant treatment in that window
b Using publicly available data on ART cycles delivered in Australia in 2022 and the number of relevant Medicare claims in the same year, we estimate approximately 92% of ART cycles delivered in Australia are captured in Medicare records.[1,2]
c Our analysis of reproductive medicines was restricted to those not classified as Section 100 fertility drugs by the Pharmaceutical Benefits Scheme, as the recording of dispensations for these drugs was handled by Services Australia for most of the study period and not present in the supplied data. Prior to collection of medicines under the copayment threshold in May 2012, Clomiphene Citrate was under the MBS copayment threshold from 1 Jan 2009 to 30 Jun 2009 and 1 Jan 2012 to 30 Apr 2012, leading to no ascertainment during these periods.

**References**


1. Australian Government Services Australia. Medicare Item Reports. Updated 26 Feb 2025. Accessed 6 Mar 2025, http://medicarestatistics.humanservices.gov.au/statistics/mbs_item.jsp
2. Newman JE, Kotevski DP, Paul RC, Chambers GM. Assisted reproductive technology in Australia and New Zealand 2022. *Sydney: National Perinatal Epidemiology and Statistics Unit, the University of New South Wales, Sydney*. 2024:1-91.


© 2026 Vajdic CM et al. *JAMA Network Open*.

**eTable 3.** ***International Classification of Diseases for Oncology*** **(ICD-O) Codes Used to Classify Incident Cancers**

| ICD-O-3.2 topography codes | ICD-O-3.2 morphology codes (behaviour code 3 except in final two rows) | Cancer type or group |
|---|---|---|
| C00-C14 | All | Lip, oral cavity, pharynx |
| C15 | All | Esophagus |
| C16 | All | Stomach |
| C17 | All | Small intestine |
| C18 | All | Colon |
| C19-C20 | All | Rectum |
| C18-C20 | All | Colorectal (excluding anus) |
| C21 | All | Anus, anal canal |
| C18-C21 | All | Colorectal (including anus) |
| C22 | All | Liver |
| C23-C24 | All | Gallbladder and other biliary tract |
| C25 | All | Pancreas |
| C26 | All | Other digestive organs |
| C30-C31 | All | Nasal cavity (nasal cavity, accessory sinuses) |
| C32 | All | Larynx |
| C33-C34 | All | Trachea, bronchus, lung |
| C37-C39 | All | Other thoracic and respiratory organs |
| C40-C41 | All | Bone, articular cartilage |
| C44, C80 | 872-879 | Melanoma of skin (invasive) |
| C44, C51, C80 | 872-879 | Melanoma of skin including genitals (invasive) |
| All | 872-879 | Melanoma (invasive) |
| C44 | All excluding 872-879 | Skin cancer (excluding melanoma) |
| All | 905 | Mesothelioma |
| All | 9140 | Kaposi sarcoma |
| C48 | All | Peritoneum |
| C48 excluding C48.1, C48.2 | All | Peritoneum excluding likely ovarian |
| C47, C49 | All | Connective and soft tissue |
| C50 | All | Breast |
| C50 | All excluding 8714, 8800-8831, 8840-8850, 8852-8921, 8930, 8935, 8990-8991, 9040-9044, 9120-9133, 9150, 9170, 9180-9243, 9260, 9364-9368, 9540-9581 | Breast excluding sarcoma |
| C51 | All | Vulva |
| C52 | All | Vagina |
| C53 | All | Cervix |
| C54-C55 | All | Uterus |
| C54-C55 | 8380, 8381, 8382, 8383 | Uterus endometrioid adenocarcinoma |
| C54-C55 | 8140 | Uterus adenocarcinoma NOS |
| C54-C55 | 8560, 8570 | Uterus adenocarcinoma with squamous differentiation |
| C54-C55 | 8441, 8460, 8461 | Uterus serous/papillary serous |
| C54-C55 | 8323 | Uterus mixed cell adenocarcinoma |
| C54-C55 | 8310 | Uterus clear cell |
| C54-C55 | 8480, 8481, 8482 | Uterus mucinous adenocarcinoma |
| C54-C55 | 8714, 8800-8806, 8810-8815, 8825, 8890-8896, 8900-8902, 8910-8912, 8930-8935, 8990-8991 | Uterus sarcoma (excluding carcinosarcoma) |
| C54-C55 | 8380, 8381, 8382, 8383, 8140, 8560, 8570 | Uterus type I (endometrioid carcinoma, adenocarcinoma NOS, adenocarcinoma with squamous differentiation) |
| C54-C55 | 8441, 8460, 8461, 8323 | Uterus type II (serous/papillary serous and mixed cell adenocarcinoma) |
| C56, C57.0, C48.1, C48.2 | All | Ovary |
| C56, C57.0, C48.1, C48.2 | 8020, 8021, 8022, 8050, 8120, 8130, 8260, 8441, 8442, 8450, 8460-8463, 9014 | Ovary serous (excluding overlapping lesion of the female genital organs) |

© 2026 Vajdic CM et al. *JAMA Network Open*.

| ICD-O-3.2 topography codes | ICD-O-3.2 morphology codes (behaviour code 3 except in final two rows) | Cancer type or group |
|---|---|---|
| C56, C57.0, C57.8, C48.1, C48.2 | 8020, 8021, 8022, 8050, 8120, 8130, 8260, 8441, 8442, 8450, 8460-8463, 9014 | Ovary serous (including overlapping lesion of the female genital organs) |
| C56, C57.0, C48.1, C48.2 | All except 8020, 8021, 8022, 8050, 8120, 8130, 8260, 8441, 8442, 8450, 8460-8463, 9014 | Ovary non-serous |
| C56, C57.0, C48.1, C48.2 | 8380-8383, 8482, 8570 | Ovary endometrioid |
| C56, C57.0, C48.1, C48.2 | 8470, 8471, 8472, 8480, 8481, 9015 | Ovary mucinous |
| C56, C57.0, C48.1, C48.2 | 8290, 8310, 8313, 8443, 8444 | Ovary clear cell |
| C56, C57.0, C48.1, C48.2 | All morphology codes other than those listed above for serous, endometrioid, mucinous, and clear cell | Ovary other and non-specified |
| C57-C58, excluding C57.0 | All | Other female genital organs and placenta |
| C64 | All | Kidney |
| C67 | All | Bladder |
| C65-C66, C68 | All | Other urinary organs |
| C69 | All | Eye |
| C71 | All | Brain |
| C70, C72 | All | Other CNS |
| C70-C72, C75.1-C75.3 | All | All CNS |
| C73 | All | Thyroid |
| C74-C75 excluding C75.1-C75.3 | All | Other endocrine glands |
| C80 | All | Unknown primary |
| All | 959-999 | All lymphoid, hematopoietic |
| All | 965-966 | Hodgkin lymphoma |
| All | 9591, 9596-9597, 9671, 9673, 9675, 9678-9680, 9684, 9687-9691, 9695, 9698-9702, 9705, 9708-9709, 9712, 9714-9719, 9724, 9726, 9727 (years 1982-2010), 9735, 9737-9738, 9761, 9764, 9766 | Non-Hodgkin lymphoma |
| All | 9596-9597, 9671, 9673, 9675, 9678-9680, 9684, 9687-9691, 9695, 9698-9699, 9712, 9731-9735, 9737-9738, 9761-9762, 9764, 9766, 9811-9819, 9823, 9833, 9940 | B-cell neoplasms |
| All | 9700-9702, 9705, 9708-9709, 9714-9719, 9724, 9726, 9768, 9827, 9831, 9834, 9837, 9948 | T-cell and NK-cell neoplasms |
| All | 9731-9734 | Plasma cell neoplasms |
| All | 980-994, 9984 | All leukemias |
| All | 9727 (years 2011+), 9840, 9861, 9865-9867, 9869-9874, 9877-9879, 9891, 9895-9898, 9910-9912, 9920, 9930-9931, 9984 | Acute myeloid leukemia |
| All | 9727 (years 1982-2010), 9811-9819, 9835, 9837 | Acute lymphoblastic leukemia |
| All | 9863, 9875 | Chronic myeloid leukemia |
| C44 | 872-879, behaviour code 2 | Melanoma in situ of skin |
| C50 | All, behaviour code 2 | Carcinoma in situ of breast |

ICD-O: International classification of Diseases for Oncology

© 2026 Vajdic CM et al. *JAMA Network Open*.

## eTable 4. SIRs and Rate Differences for All Cancers for Those Exposed to Medically Assisted Reproduction
## eTable 4a. SIRs and rate difference (per 100,000 person-years) for all cancers for those who received assisted reproductive therapy treatments (ART)

| Cancer type | ICD-O3.2 topographies | ICD-O3.2 morphologies[a] | Observed cancers | Expected cancers | SIR (95% CI) | Rate difference (95% CI) |
|---|---|---|---|---|---|---|
| All invasive cancers | All | All | 10052 | 10079.38 | 1.00 (0.98, 1.02) | -0.91 (-7.38, 5.66) |
| Lip, oral cavity, and pharynx | C00-C14 | All | 157 | 179.35 | 0.88 (0.74, 1.02) | -0.74 (-1.52, 0.14) |
| Esophagus | C15 | All | 20 | 23.67 | 0.84 (0.52, 1.30) | -0.12 (-0.38, 0.24) |
| Stomach | C16 | All | 85 | 90.72 | 0.94 (0.75, 1.16) | -0.19 (-0.76, 0.48) |
| Small intestine | C17 | All | 53 | 40.19 | 1.32 (0.99, 1.73) | 0.42 (-0.02, 0.96) |
| Colorectum (excluding anus) | C18-C20 | All | 713 | 726.31 | 0.98 (0.91, 1.06) | -0.44 (-2.14, 1.36) |
| Anus | C21 | All | 35 | 45.5 | 0.77 (0.54, 1.07) | -0.35 (-0.70, 0.11) |
| Liver | C22 | All | 43 | 51.08 | 0.84 (0.61, 1.13) | -0.27 (-0.66, 0.23) |
| Gallbladder | C23-C24 | All | 21 | 30.67 | 0.68 (0.42, 1.05) | -0.32 (-0.59, 0.05) |
| Pancreas | C25 | All | 79 | 105.6 | 0.75 (0.59, 0.93) | -0.88 (-1.43, -0.24) |
| Other digestive | C26 | All | * | * | * | * |
| Nasal cavity | C30-C31 | All | 10 | 12.57 | 0.80 (0.38, 1.46) | -0.08 (-0.26, 0.19) |
| Larynx | C32 | All | * | * | * | * |
| Trachea, bronchus, and lung | C33-C34 | All | 268 | 383.47 | 0.70 (0.62, 0.79) | -3.82 (-4.85, -2.69) |
| Other respiratory | C37-C39 | All | 25 | 11.87 | 2.11 (1.36, 3.11) | 0.43 (0.14, 0.83) |
| Bone | C40-C41 | All | 23 | 18.99 | 1.21 (0.77, 1.82) | 0.13 (-0.15, 0.51) |
| Melanoma (invasive) | All | 872-879 | 1407 | 1314.23 | 1.07 (1.02, 1.13) | 3.07 (0.67, 5.57) |
| Melanoma (in situ) | C44 | 872-879, behaviour code 2 | 913 | 771.15 | 1.18 (1.11, 1.26) | 8.92 (5.26, 12.78) |
| Skin (excluding melanoma) | C44 | All excluding 872-879 | 37 | 19.87 | 1.86 (1.31, 2.57) | 0.57 (0.20, 1.03) |
| Mesothelioma | All | 905 | 11 | 8.71 | 1.26 (0.63, 2.26) | 0.08 (-0.11, 0.36) |
| Kaposi sarcoma | All | 9140 | 0 | 0.55 | 0.00 (0.00, 6.76) | -0.02 (-0.02, 0.10) |
| Peritoneum[c] | C48 excluding C48.1, C48.2 | All | 12 | 7.65 | 1.57 (0.81, 2.74) | 0.14 (-0.05, 0.44) |
| Connective tissue | C47, C49 | All | 67 | 60.54 | 1.11 (0.86, 1.41) | 0.21 (-0.29, 0.81) |
| Breast (invasive) | C50 | All | 4032 | 3936.58 | 1.02 (0.99, 1.06) | 3.16 (-0.93, 7.34) |

© 2026 Vajdic CM et al. *JAMA Network Open*.

| Cancer type | ICD-O3.2 topographies | ICD-O3.2 morphologies[a] | Observed cancers | Expected cancers | SIR (95% CI) | Rate difference (95% CI) |
|---|---|---|---|---|---|---|
| Breast (in situ) | C50 | All, behaviour code 2 | 368 | 296.26 | 1.24 (1.12, 1.38) | 3.94 (1.93, 6.11) |
| Vulva | C51 | All | 35 | 53.93 | 0.65 (0.45, 0.90) | -0.63 (-0.98, -0.17) |
| Vagina | C52 | All | 10 | 11.03 | 0.91 (0.43, 1.67) | -0.03 (-0.21, 0.24) |
| Cervix | C53 | All | 217 | 355.95 | 0.61 (0.53, 0.70) | -4.60 (-5.52, -3.58) |
| Uterus | C54-C55 | All | 476 | 388.11 | 1.23 (1.12, 1.34) | 2.91 (1.53, 4.39) |
| Ovary | C56, C57.0, C48.1, C48.2 | All | 330 | 269.19 | 1.23 (1.10, 1.37) | 2.01 (0.87, 3.26) |
| Other genital and placenta | C57-C58, excluding C57.0 | All | 22 | 15.27 | 1.44 (0.90, 2.18) | 0.22 (-0.05, 0.60) |
| Kidney | C64 | All | 130 | 180.18 | 0.72 (0.60, 0.86) | -1.66 (-2.37, -0.85) |
| Bladder | C67 | All | 17 | 30.88 | 0.55 (0.32, 0.88) | -0.46 (-0.69, -0.12) |
| Other urinary | C65-C66, C68 | All | * | * | * | * |
| Eye | C69 | All | 23 | 22.01 | 1.05 (0.66, 1.57) | 0.03 (-0.25, 0.41) |
| Brain | C71 | All | 116 | 126.88 | 0.91 (0.76, 1.10) | -0.36 (-1.03, 0.41) |
| Other CNS | C70, C72 | All | 11 | 10.12 | 1.09 (0.54, 1.95) | 0.03 (-0.15, 0.32) |
| Thyroid | C73 | All | 736 | 702.13 | 1.05 (0.97, 1.13) | 1.12 (-0.61, 2.95) |
| Other endocrine glands | C74-C75 excluding C75.1-C75.3 | All | 10 | 12.42 | 0.81 (0.39, 1.48) | -0.08 (-0.25, 0.20) |
| Unknown primary | C80 | All | 60 | 78.37 | 0.77 (0.58, 0.99) | -0.61 (-1.08, -0.04) |
| Hematological | All | 959-999 | 740 | 733.06 | 1.01 (0.94, 1.08) | 0.23 (-1.50, 2.06) |

SIR: Standardised incidence ratio
a Behaviour code 3 unless otherwise specified
b Cancers with fewer than 6 observed cancers censored to preserve privacy
c Excluding sites coded to peritoneum that are likely primary ovarian cancers: ICD-O3.2 topography 8.1, C48.2

© 2026 Vajdic CM et al. *JAMA Network Open*.

**eTable 4b. SIRs and rate difference (per 100,000 person-years) for all cancers for those who received intrauterine insemination with ovarian stimulation**

| Cancer type | ICD-O3.2 topographies | ICD-O3.2 morphologies[a] | Observed cancers | Expected cancers | SIR (95% CI) | Rate difference (95% CI) |
|---|---|---|---|---|---|---|
| All invasive cancers | All | All | 5290 | 5316.75 | 0.99 (0.97, 1.02) | -1.75 (-10.99, 7.69) |
| Lip, oral cavity, and pharynx | C00-C14 | All | 82 | 96.11 | 0.85 (0.68, 1.06) | -0.92 (-2.02, 0.37) |
| Esophagus | C15 | All | 8 | 13.27 | 0.60 (0.26, 1.19) | -0.34 (-0.64, 0.16) |
| Stomach | C16 | All | 55 | 48.18 | 1.14 (0.86, 1.49) | 0.45 (-0.44, 1.53) |
| Small intestine | C17 | All | 26 | 21.48 | 1.21 (0.79, 1.77) | 0.30 (-0.29, 1.09) |
| Colorectum (excluding anus) | C18-C20 | All | 350 | 382.3 | 0.92 (0.82, 1.02) | -2.11 (-4.44, 0.42) |
| Anus | C21 | All | 16 | 24.79 | 0.65 (0.37, 1.05) | -0.57 (-1.02, 0.08) |
| Liver | C22 | All | 25 | 27.53 | 0.91 (0.59, 1.34) | -0.17 (-0.74, 0.61) |
| Gallbladder | C23-C24 | All | 9 | 16.91 | 0.53 (0.24, 1.01) | -0.52 (-0.84, 0.01) |
| Pancreas | C25 | All | 42 | 57.01 | 0.74 (0.53, 1.00) | -0.98 (-1.75, -0.02) |
| Other digestive | C26 | All | * | * | * | * |
| Nasal cavity | C30-C31 | All | * | * | * | * |
| Larynx | C32 | All | * | * | * | * |
| Trachea, bronchus, and lung | C33-C34 | All | 131 | 213 | 0.62 (0.51, 0.73) | -5.35 (-6.76, -3.76) |
| Other respiratory | C37-C39 | All | 14 | 6.43 | 2.18 (1.19, 3.65) | 0.49 (0.08, 1.11) |
| Bone | C40-C41 | All | 6 | 9.73 | 0.62 (0.23, 1.34) | -0.24 (-0.49, 0.22) |
| Melanoma (invasive) | All | 872-8790 | 790 | 719.42 | 1.10 (1.02, 1.18) | 4.61 (1.07, 8.34) |
| Melanoma (in situ) | C44 | 872-879, behaviour code 2 | 402 | 338.68 | 1.19 (1.07, 1.31) | 9.87 (3.89, 16.31) |
| Skin (excluding melanoma) | C44 | All excluding 872-879 | 26 | 11.03 | 2.36 (1.54, 3.45) | 0.98 (0.39, 1.77) |
| Mesothelioma | All | 905 | 6 | 4.84 | 1.24 (0.46, 2.70) | 0.08 (-0.17, 0.54) |
| Kaposi sarcoma | All | 9140 | 0 | 0.29 | 0.00 (0.00, 12.58) | -0.02 (-0.02, 0.22) |
| Peritoneum[c] | C48 excluding C48.1, C48.2 | All | * | * | * | * |
| Connective tissue | C47, C49 | All | 29 | 30.75 | 0.94 (0.63, 1.35) | -0.11 (-0.74, 0.71) |
| Breast (invasive) | C50 | All | 2113 | 2055.52 | 1.03 (0.98, 1.07) | 3.75 (-2.07, 9.77) |

© 2026 Vajdic CM et al. *JAMA Network Open*.

| Cancer type | ICD-O3.2 topographies | ICD-O3.2 morphologies[a] | Observed cancers | Expected cancers | SIR (95% CI) | Rate difference (95% CI) |
|---|---|---|---|---|---|---|
| Breast (in situ) | C50 | All, behaviour code 2 | 145 | 127.22 | 1.14 (0.96, 1.34) | 2.32 (-0.63, 5.66) |
| Vulva | C51 | All | 18 | 28.56 | 0.63 (0.37, 1.00) | -0.69 (-1.17, -0.01) |
| Vagina | C52 | All | * | * | * | * |
| Cervix | C53 | All | 94 | 181.32 | 0.52 (0.42, 0.63) | -5.70 (-6.88, -4.33) |
| Uterus | C54-C55 | All | 272 | 206.24 | 1.32 (1.17, 1.49) | 4.29 (2.25, 6.54) |
| Ovary | C56, C57.0, C48.1, C48.2 | All | 167 | 141.34 | 1.18 (1.01, 1.37) | 1.68 (0.08, 3.46) |
| Other genital and placenta | C57-C58, excluding C57.0 | All | 8 | 7.74 | 1.03 (0.45, 2.04) | 0.02 (-0.28, 0.52) |
| Kidney | C64 | All | 83 | 95.62 | 0.87 (0.69, 1.08) | -0.82 (-1.93, 0.47) |
| Bladder | C67 | All | 12 | 17.21 | 0.70 (0.36, 1.22) | -0.34 (-0.72, 0.25) |
| Other urinary | C65-C66, C68 | All | * | * | * | * |
| Eye | C69 | All | 15 | 11.89 | 1.26 (0.71, 2.08) | 0.20 (-0.23, 0.84) |
| Brain | C71 | All | 53 | 66.32 | 0.80 (0.60, 1.05) | -0.87 (-1.74, 0.20) |
| Other CNS | C70, C72 | All | * | * | * | * |
| Thyroid | C73 | All | 361 | 353.96 | 1.02 (0.92, 1.13) | 0.46 (-1.91, 3.02) |
| Other endocrine glands | C74-C75 excluding C75.1-C75.3 | All | 7 | 6.84 | 1.02 (0.41, 2.11) | 0.01 (-0.26, 0.50) |
| Unknown primary | C80 | All | 35 | 44.1 | 0.79 (0.55, 1.10) | -0.59 (-1.29, 0.30) |
| Hematological | All | 959-999 | 418 | 383.79 | 1.09 (0.99, 1.20) | 2.23 (-0.32, 4.98) |

SIR: Standardised incidence ratio
a Behaviour code 3 unless otherwise specified
b Cancers with fewer than 6 observed cancers censored to preserve privacy
c Excluding sites coded to peritoneum that are likely primary ovarian cancers: ICD-O3.2 topography 8.1, C48.2

© 2026 Vajdic CM et al. *JAMA Network Open*.

**eTable 4c. SIRs and rate difference (per 100,000 person-years) for all cancers for those dispensed Clomiphene Citrate**

| Cancer type | ICD-O3.2 topographies | ICD-O3.2 morphologies[a] | Observed cancers | Expected cancers | SIR (95% CI) | Rate difference (95% CI) |
|---|---|---|---|---|---|---|
| All invasive cancers | All | All | 4323 | 4176.35 | 1.04 (1.00, 1.07) | 8.61 (1.10, 16.30) |
| Lip, oral cavity, and pharynx | C00-C14 | All | 66 | 70.59 | 0.94 (0.72, 1.19) | -0.27 (-1.15, 0.79) |
| Esophagus | C15 | All | * | * | * | * |
| Stomach | C16 | All | 31 | 34.91 | 0.89 (0.60, 1.26) | -0.23 (-0.81, 0.53) |
| Small intestine | C17 | All | 20 | 15.88 | 1.26 (0.77, 1.94) | 0.24 (-0.22, 0.88) |
| Colorectum (excluding anus) | C18-C20 | All | 288 | 289.36 | 1.00 (0.88, 1.12) | -0.08 (-1.98, 1.99) |
| Anus | C21 | All | 16 | 15.67 | 1.02 (0.58, 1.66) | 0.02 (-0.38, 0.61) |
| Liver | C22 | All | 15 | 16.46 | 0.91 (0.51, 1.50) | -0.09 (-0.47, 0.49) |
| Gallbladder | C23-C24 | All | * | * | * | * |
| Pancreas | C25 | All | 31 | 34.96 | 0.89 (0.60, 1.26) | -0.23 (-0.82, 0.53) |
| Other digestive | C26 | All | * | * | * | * |
| Nasal cavity | C30-C31 | All | * | * | * | * |
| Larynx | C32 | All | * | * | * | * |
| Trachea, bronchus, and lung | C33-C34 | All | 75 | 108.83 | 0.69 (0.54, 0.86) | -1.99 (-2.93, -0.87) |
| Other respiratory | C37-C39 | All | 16 | 5.75 | 2.78 (1.59, 4.52) | 0.60 (0.20, 1.19) |
| Bone | C40-C41 | All | 13 | 10.28 | 1.26 (0.67, 2.16) | 0.16 (-0.20, 0.70) |
| Melanoma (invasive) | All | 872-8790 | 705 | 612.9 | 1.15 (1.07, 1.24) | 5.41 (2.41, 8.58) |
| Melanoma (in situ) | C44 | 872-879, behaviour code 2 | 687 | 586.71 | 1.17 (1.08, 1.26) | 7.39 (3.68, 11.32) |
| Skin (excluding melanoma) | C44 | All excluding 872-879 | 22 | 7.42 | 2.96 (1.86, 4.49) | 0.86 (0.37, 1.52) |
| Mesothelioma | All | 905 | * | * | * | * |
| Kaposi sarcoma | All | 9140 | 0 | 0.23 | 0.00 (0.00, 16.20) | -0.01 (-0.01, 0.20) |
| Peritoneum[c] | C48 excluding C48.1, C48.2 | All | * | * | * | * |
| Connective tissue | C47, C49 | All | 30 | 28.94 | 1.04 (0.70, 1.48) | 0.06 (-0.51, 0.82) |
| Breast (invasive) | C50 | All | 1527 | 1543.08 | 0.99 (0.94, 1.04) | -0.94 (-5.39, 3.67) |
| Breast (in situ) | C50 | All, behaviour code 2 | 236 | 224.57 | 1.05 (0.92, 1.19) | 0.67 (-1.04, 2.56) |

© 2026 Vajdic CM et al. *JAMA Network Open*.

| Cancer type | ICD-O3.2 topographies | ICD-O3.2 morphologies[a] | Observed cancers | Expected cancers | SIR (95% CI) | Rate difference (95% CI) |
|---|---|---|---|---|---|---|
| Vulva | C51 | All | 19 | 21.43 | 0.89 (0.53, 1.38) | -0.14 (-0.59, 0.48) |
| Vagina | C52 | All | * | * | * | * |
| Cervix | C53 | All | 123 | 203.32 | 0.60 (0.50, 0.72) | -4.72 (-5.94, -3.32) |
| Uterus | C54-C55 | All | 245 | 134.17 | 1.83 (1.60, 2.07) | 6.51 (4.76, 8.43) |
| Ovary | C56, C57.0, C48.1, C48.2 | All | 105 | 105.37 | 1.00 (0.82, 1.21) | -0.02 (-1.14, 1.28) |
| Other genital and placenta | C57-C58, excluding C57.0 | All | 7 | 6.95 | 1.01 (0.40, 2.07) | 0.00 (-0.24, 0.44) |
| Kidney | C64 | All | 61 | 69.57 | 0.88 (0.67, 1.13) | -0.50 (-1.35, 0.52) |
| Bladder | C67 | All | * | * | * | * |
| Other urinary | C65-C66, C68 | All | * | * | * | * |
| Eye | C69 | All | 13 | 8.72 | 1.49 (0.79, 2.55) | 0.25 (-0.11, 0.79) |
| Brain | C71 | All | 66 | 58.93 | 1.12 (0.87, 1.42) | 0.42 (-0.46, 1.47) |
| Other CNS | C70, C72 | All | * | * | * | * |
| Thyroid | C73 | All | 446 | 374.65 | 1.19 (1.08, 1.31) | 4.19 (1.82, 6.74) |
| Other endocrine glands | C74-C75 excluding C75.1-C75.3 | All | * | * | * | * |
| Unknown primary | C80 | All | 22 | 25.76 | 0.85 (0.54, 1.29) | -0.22 (-0.70, 0.44) |
| Hematological | All | 959-999 | 325 | 313.16 | 1.04 (0.93, 1.16) | 0.70 (-1.32, 2.89) |

SIR: Standardised incidence ratio
a Behaviour code 3 unless otherwise specified
b Cancers with fewer than 6 observed cancers censored to preserve privacy
c Excluding sites coded to peritoneum that are likely primary ovarian cancers: ICD-O3.2 topography 8.1, C48.2

© 2026 Vajdic CM et al. *JAMA Network Open*.

**eFigure 1. Forest Plot of SIRs and Rate Differences (Per 100 000 Person-Years) for Invasive Breast Cancer by MAR Cohort and Time-Varying Woman and Treatment Characteristics**

| | | Observed cancers in cohort | SIR | Rate difference |
|---|---|---|---|---|
| **Attained age group** | | | | |
| 18-35 | ART | 100 | 0.92 (0.74, 1.11) | -2.23 (-6.74, 3.00) |
| 35-39 | ART | 398 | 0.92 (0.83, 1.02) | -4.73 (-10.14, 1.10) |
| 40-44 | ART | 926 | 0.96 (0.90, 1.03) | -4.73 (-12.41, 3.33) |
| 45-49 | ART | 1176 | 1.05 (0.99, 1.12) | 10.82 (-1.25, 23.42) |
| >50 | ART | 1432 | 1.09 (1.03, 1.14) | 19.47 (7.05, 32.38) |
| 18-35 | IUI/OS | 51 | 0.93 (0.69, 1.22) | -1.84 (-8.09, 5.86) |
| 35-39 | IUI/OS | 195 | 0.94 (0.82, 1.09) | -3.42 (-11.24, 5.27) |
| 40-44 | IUI/OS | 433 | 0.94 (0.85, 1.03) | -8.07 (-18.91, 3.56) |
| 45-49 | IUI/OS | 573 | 1.00 (0.92, 1.08) | -0.12 (-16.44, 17.25) |
| >50 | IUI/OS | 861 | 1.14 (1.06, 1.21) | 30.65 (13.81, 48.37) |
| 18-35 | Clomiphene Citrate | 109 | 1.03 (0.85, 1.25) | 0.86 (-3.85, 6.28) |
| 35-39 | Clomiphene Citrate | 265 | 0.95 (0.83, 1.07) | -3.38 (-10.17, 4.06) |
| 40-44 | Clomiphene Citrate | 454 | 0.97 (0.88, 1.06) | -4.01 (-15.09, 7.88) |
| 45-49 | Clomiphene Citrate | 430 | 1.01 (0.92, 1.11) | 1.71 (-17.60, 22.46) |
| >50 | Clomiphene Citrate | 269 | 1.03 (0.91, 1.16) | 2.70 (-9.35, 15.90) |
| **Calendar year** | | | | |
| 1991-2000 | ART | 128 | 0.95 (0.79, 1.13) | -3.31 (-13.50, 8.30) |
| 2001-2005 | ART | 269 | 0.90 (0.79, 1.01) | -9.57 (-19.44, 1.22) |
| 2006-2010 | ART | 662 | 1.02 (0.95, 1.10) | 2.49 (-6.17, 11.67) |
| 2011-2015 | ART | 1365 | 1.08 (1.02, 1.13) | 10.52 (2.70, 18.66) |
| 2016-2019 | ART | 1608 | 1.01 (0.97, 1.06) | 2.19 (-5.48, 10.16) |
| 1991-2000 | IUI/OS | 85 | 1.03 (0.82, 1.27) | 1.70 (-11.16, 16.82) |
| 2001-2005 | IUI/OS | 164 | 0.90 (0.77, 1.05) | -9.46 (-21.82, 4.42) |
| 2006-2010 | IUI/OS | 368 | 1.01 (0.91, 1.12) | 1.06 (-10.74, 13.81) |
| 2011-2015 | IUI/OS | 686 | 1.05 (0.97, 1.13) | 7.50 (-3.95, 19.62) |
| 2016-2019 | IUI/OS | 810 | 1.05 (0.98, 1.12) | 8.27 (-3.85, 21.03) |
| 2002-2005 | Clomiphene Citrate | 40 | 1.11 (0.80, 1.52) | 5.08 (-9.24, 23.22) |
| 2006-2010 | Clomiphene Citrate | 212 | 1.00 (0.87, 1.15) | 0.20 (-7.77, 9.02) |
| 2011-2015 | Clomiphene Citrate | 558 | 1.03 (0.95, 1.12) | 2.79 (-4.61, 10.67) |
| 2016-2019 | Clomiphene Citrate | 717 | 0.95 (0.88, 1.02) | -5.71 (-13.48, 2.49) |
| **Any children** | | | | |
| Nulliparous | ART | 2169 | 1.01 (0.97, 1.05) | 1.47 (-4.65, 7.79) |
| Parous | ART | 1782 | 1.05 (1.00, 1.10) | 5.72 (0.22, 11.42) |
| Nulliparous | IUI/OS | 1252 | 1.05 (1.00, 1.12) | 8.12 (-0.40, 17.00) |
| Parous | IUI/OS | 790 | 0.98 (0.92, 1.05) | -1.97 (-9.80, 6.29) |
| Nulliparous | Clomiphene Citrate | 508 | 0.95 (0.87, 1.03) | -5.28 (-13.33, 3.31) |
| Parous | Clomiphene Citrate | 957 | 1.01 (0.94, 1.07) | 0.66 (-4.66, 6.25) |
| **Number of treatments** | | | | |
| 1-2 | ART | 2121 | 0.99 (0.95, 1.03) | -1.57 (-6.58, 3.60) |
| 3-5 | ART | 1281 | 1.09 (1.03, 1.15) | 11.94 (3.82, 20.40) |
| 6+ | ART | 630 | 1.04 (0.96, 1.12) | 5.63 (-6.90, 18.93) |
| 1-2 | IUI/OS | 1329 | 1.00 (0.95, 1.06) | 0.35 (-6.62, 7.61) |
| 3-5 | IUI/OS | 552 | 1.10 (1.01, 1.20) | 13.51 (1.40, 26.40) |
| 6+ | IUI/OS | 232 | 1.02 (0.89, 1.16) | 2.45 (-17.11, 24.02) |
| 1-2 | Clomiphene Citrate | 960 | 1.01 (0.95, 1.07) | 0.79 (-4.84, 6.70) |
| 3-5 | Clomiphene Citrate | 422 | 0.98 (0.89, 1.07) | -2.17 (-10.45, 6.73) |
| 6+ | Clomiphene Citrate | 145 | 0.91 (0.77, 1.07) | -8.57 (-22.21, 6.87) |
| **Years since first treatment** | | | | |
| <1 | ART | 113 | 0.74 (0.61, 0.89) | -14.15 (-21.38, -5.82) |
| 1-2 | ART | 286 | 0.82 (0.73, 0.93) | -11.89 (-18.18, -5.02) |
| 3-5 | ART | 590 | 1.00 (0.92, 1.08) | 0.07 (-7.19, 7.78) |
| 6+ | ART | 3043 | 1.07 (1.03, 1.11) | 12.23 (5.50, 19.15) |
| <1 | IUI/OS | 42 | 0.65 (0.47, 0.87) | -19.10 (-28.82, -6.86) |
| 1-2 | IUI/OS | 137 | 0.91 (0.76, 1.08) | -5.89 (-15.48, 5.00) |
| 3-5 | IUI/OS | 243 | 0.92 (0.81, 1.05) | -6.89 (-16.77, 3.98) |
| 6+ | IUI/OS | 1691 | 1.07 (1.02, 1.13) | 13.00 (3.97, 22.37) |
| <1 | Clomiphene Citrate | 54 | 0.76 (0.57, 0.99) | -9.69 (-17.35, -0.31) |
| 1-2 | Clomiphene Citrate | 183 | 1.08 (0.93, 1.25) | 4.17 (-3.46, 12.68) |
| 3-5 | Clomiphene Citrate | 292 | 0.96 (0.85, 1.07) | -3.11 (-10.60, 5.06) |
| 6+ | Clomiphene Citrate | 998 | 1.00 (0.94, 1.06) | 0.06 (-7.99, 8.50) |

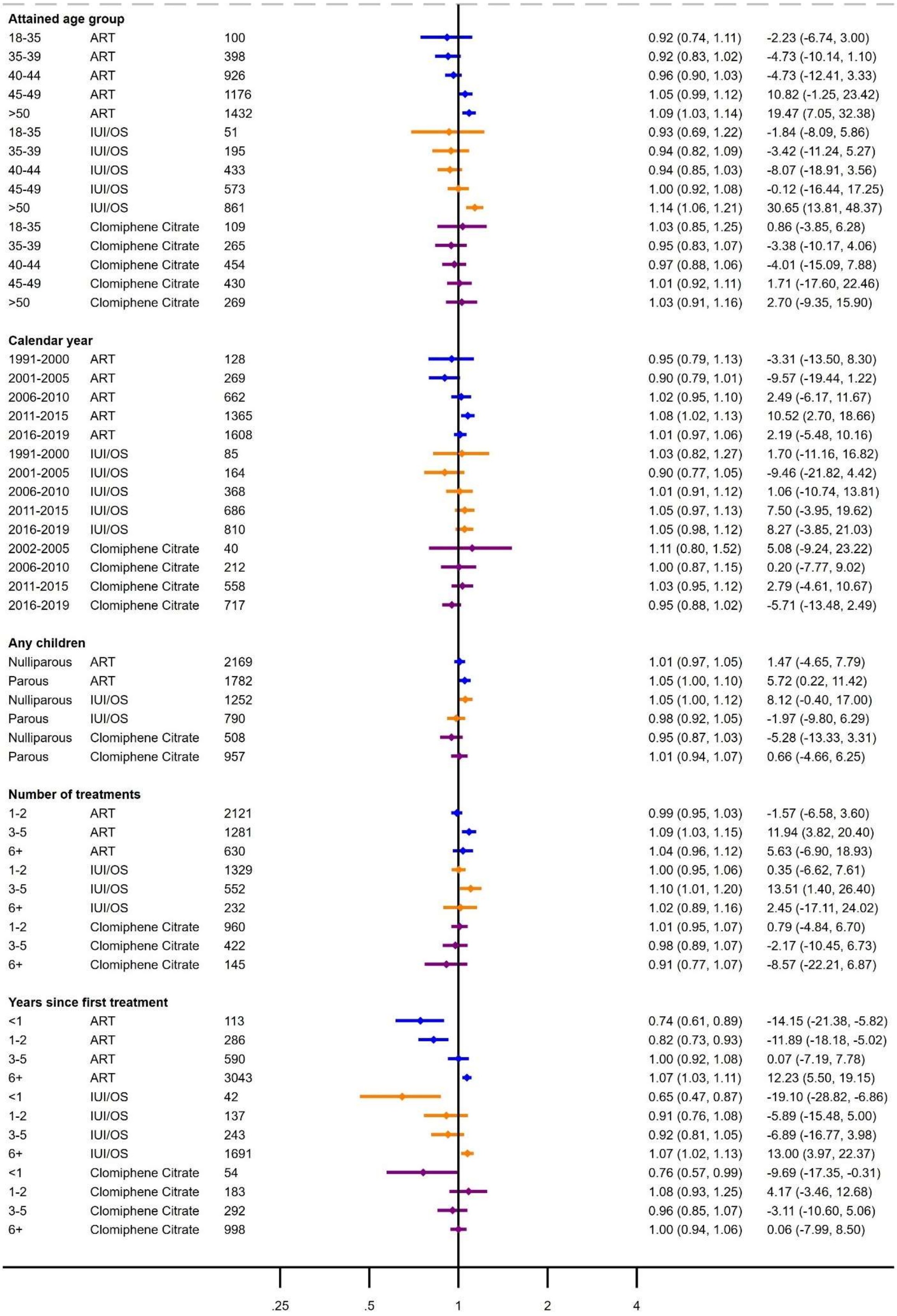


© 2026 Vajdic CM et al. *JAMA Network Open*.

**eFigure 2. Forest Plot of SIRs and Rate Differences (Per 100 000 Person-Years) for In-Situ Breast Cancer by MAR Cohort and Time-Varying Woman and Treatment Characteristics**

| | | Observed cancers in cohort | SIR | Rate difference |
|---|---|---|---|---|
| **Attained age group** | | | | |
| 40-44 | ART | 124 | 1.32 (1.10, 1.58) | 5.84 (1.80, 10.46) |
| 45-49 | ART | 124 | 1.24 (1.03, 1.48) | 7.99 (1.11, 15.85) |
| >50 | ART | 91 | 1.42 (1.14, 1.74) | 13.58 (4.62, 24.06) |
| 40-44 | IUI/OS | 48 | 1.20 (0.89, 1.59) | 3.81 (-2.17, 11.23) |
| 45-49 | IUI/OS | 42 | 0.99 (0.71, 1.33) | -0.42 (-9.67, 11.22) |
| >50 | IUI/OS | 33 | 1.15 (0.80, 1.62) | 4.97 (-6.57, 19.93) |
| 18-35 | Clomiphene Citrate | 7 | 0.90 (0.36, 1.86) | -0.18 (-1.19, 1.61) |
| 35-39 | Clomiphene Citrate | 32 | 1.08 (0.74, 1.53) | 0.54 (-1.68, 3.43) |
| 40-44 | Clomiphene Citrate | 76 | 1.11 (0.87, 1.39) | 2.00 (-2.38, 7.20) |
| 45-49 | Clomiphene Citrate | 65 | 0.95 (0.73, 1.21) | -1.61 (-8.83, 7.08) |
| >50 | Clomiphene Citrate | 56 | 1.11 (0.84, 1.44) | 2.18 (-3.11, 8.64) |
| **Calendar year** | | | | |
| 2011-2015 | ART | 122 | 1.21 (1.00, 1.44) | 3.20 (0.03, 6.82) |
| 2016-2019 | ART | 222 | 1.37 (1.20, 1.56) | 7.46 (3.95, 11.34) |
| 2006-2010 | IUI/OS | 13 | 0.94 (0.50, 1.60) | -0.60 (-4.84, 5.84) |
| 2011-2015 | IUI/OS | 58 | 1.33 (1.01, 1.73) | 5.31 (0.21, 11.51) |
| 2016-2019 | IUI/OS | 74 | 1.09 (0.85, 1.37) | 1.86 (-3.10, 7.76) |
| 2011-2015 | Clomiphene Citrate | 88 | 1.13 (0.91, 1.39) | 1.65 (-1.19, 4.98) |
| 2016-2019 | Clomiphene Citrate | 118 | 1.01 (0.84, 1.21) | 0.17 (-2.89, 3.68) |
| **Any children** | | | | |
| Nulliparous | ART | 147 | 1.19 (1.01, 1.40) | 3.52 (0.15, 7.34) |
| Parous | ART | 200 | 1.24 (1.07, 1.42) | 3.50 (1.06, 6.21) |
| Nulliparous | IUI/OS | 56 | 1.04 (0.79, 1.35) | 0.78 (-3.95, 6.55) |
| Parous | IUI/OS | 80 | 1.20 (0.95, 1.50) | 2.99 (-0.69, 7.34) |
| Nulliparous | Clomiphene Citrate | 75 | 0.90 (0.71, 1.13) | -1.51 (-4.49, 2.03) |
| Parous | Clomiphene Citrate | 148 | 1.13 (0.95, 1.32) | 1.48 (-0.56, 3.79) |
| **Number of treatments** | | | | |
| 1-2 | ART | 195 | 1.19 (1.03, 1.37) | 2.81 (0.47, 5.42) |
| 3-5 | ART | 121 | 1.38 (1.14, 1.65) | 6.77 (2.58, 11.58) |
| 6+ | ART | 52 | 1.15 (0.86, 1.51) | 3.32 (-3.10, 11.22) |
| 1-2 | IUI/OS | 102 | 1.12 (0.92, 1.37) | 2.03 (-1.34, 5.93) |
| 3-5 | IUI/OS | 33 | 1.13 (0.78, 1.58) | 2.17 (-3.81, 9.92) |
| 6+ | IUI/OS | 10 | 1.37 (0.66, 2.53) | 7.65 (-6.99, 31.24) |
| 1-2 | Clomiphene Citrate | 144 | 1.04 (0.88, 1.23) | 0.55 (-1.57, 2.96) |
| 3-5 | Clomiphene Citrate | 65 | 1.03 (0.80, 1.32) | 0.42 (-2.70, 4.18) |
| 6+ | Clomiphene Citrate | 27 | 1.15 (0.76, 1.68) | 2.14 (-3.40, 9.54) |
| **Years since first treatment** | | | | |
| <1 | ART | 10 | 0.68 (0.33, 1.25) | -2.14 (-4.48, 1.65) |
| 1-2 | ART | 32 | 0.90 (0.62, 1.28) | -0.84 (-3.33, 2.40) |
| 3-5 | ART | 74 | 1.17 (0.92, 1.47) | 2.23 (-1.05, 6.13) |
| 6+ | ART | 252 | 1.38 (1.21, 1.56) | 9.73 (5.48, 14.39) |
| 3-5 | IUI/OS | 30 | 1.16 (0.78, 1.66) | 2.12 (-2.79, 8.57) |
| 6+ | IUI/OS | 97 | 1.19 (0.97, 1.46) | 4.91 (-0.83, 11.58) |
| 3-5 | Clomiphene Citrate | 45 | 1.14 (0.83, 1.53) | 1.27 (-1.53, 4.77) |
| 6+ | Clomiphene Citrate | 173 | 1.10 (0.94, 1.27) | 2.02 (-1.26, 5.69) |

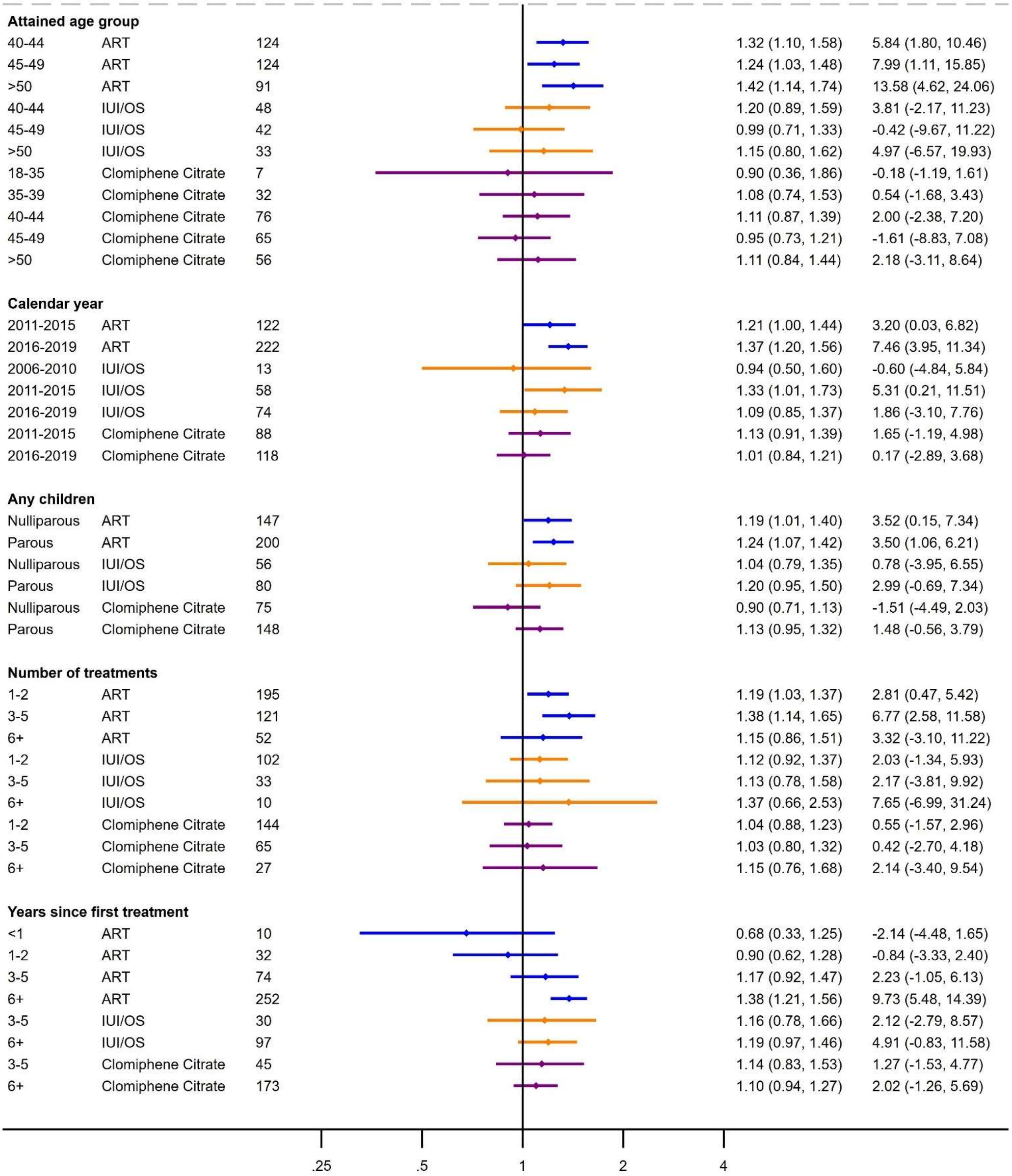


Note: Some categories had small numbers of observed cases and could not be reported.

© 2026 Vajdic CM et al. *JAMA Network Open*.

**eFigure 3. Forest Plot of SIRs and Rate Differences (Per 100 000 Person-Years) for Uterine Cancer by MAR Cohort and Time-Varying Woman and Treatment Characteristics**

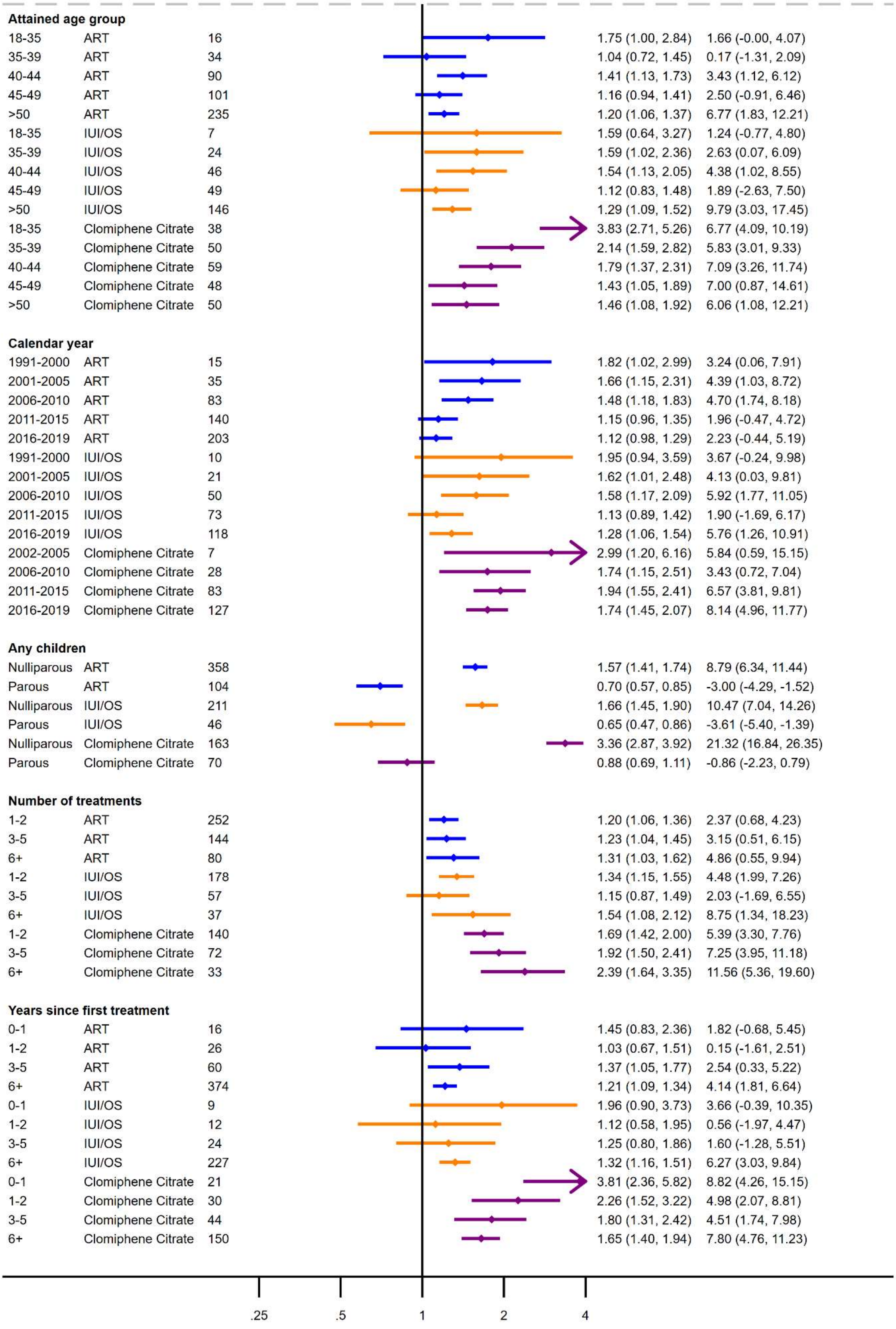


| | | Observed cancers in cohort | SIR | Rate difference |
|---|---|---|---|---|
| **Attained age group** | | | | |
| 18-35 | ART | 16 | 1.75 (1.00, 2.84) | 1.66 (-0.00, 4.07) |
| 35-39 | ART | 34 | 1.04 (0.72, 1.45) | 0.17 (-1.31, 2.09) |
| 40-44 | ART | 90 | 1.41 (1.13, 1.73) | 3.43 (1.12, 6.12) |
| 45-49 | ART | 101 | 1.16 (0.94, 1.41) | 2.50 (-0.91, 6.46) |
| >50 | ART | 235 | 1.20 (1.06, 1.37) | 6.77 (1.83, 12.21) |
| 18-35 | IUI/OS | 7 | 1.59 (0.64, 3.27) | 1.24 (-0.77, 4.80) |
| 35-39 | IUI/OS | 24 | 1.59 (1.02, 2.36) | 2.63 (0.07, 6.09) |
| 40-44 | IUI/OS | 46 | 1.54 (1.13, 2.05) | 4.38 (1.02, 8.55) |
| 45-49 | IUI/OS | 49 | 1.12 (0.83, 1.48) | 1.89 (-2.63, 7.50) |
| >50 | IUI/OS | 146 | 1.29 (1.09, 1.52) | 9.79 (3.03, 17.45) |
| 18-35 | Clomiphene Citrate | 38 | 3.83 (2.71, 5.26) | 6.77 (4.09, 10.19) |
| 35-39 | Clomiphene Citrate | 50 | 2.14 (1.59, 2.82) | 5.83 (3.01, 9.33) |
| 40-44 | Clomiphene Citrate | 59 | 1.79 (1.37, 2.31) | 7.09 (3.26, 11.74) |
| 45-49 | Clomiphene Citrate | 48 | 1.43 (1.05, 1.89) | 7.00 (0.87, 14.61) |
| >50 | Clomiphene Citrate | 50 | 1.46 (1.08, 1.92) | 6.06 (1.08, 12.21) |
| **Calendar year** | | | | |
| 1991-2000 | ART | 15 | 1.82 (1.02, 2.99) | 3.24 (0.06, 7.91) |
| 2001-2005 | ART | 35 | 1.66 (1.15, 2.31) | 4.39 (1.03, 8.72) |
| 2006-2010 | ART | 83 | 1.48 (1.18, 1.83) | 4.70 (1.74, 8.18) |
| 2011-2015 | ART | 140 | 1.15 (0.96, 1.35) | 1.96 (-0.47, 4.72) |
| 2016-2019 | ART | 203 | 1.12 (0.98, 1.29) | 2.23 (-0.44, 5.19) |
| 1991-2000 | IUI/OS | 10 | 1.95 (0.94, 3.59) | 3.67 (-0.24, 9.98) |
| 2001-2005 | IUI/OS | 21 | 1.62 (1.01, 2.48) | 4.13 (0.03, 9.81) |
| 2006-2010 | IUI/OS | 50 | 1.58 (1.17, 2.09) | 5.92 (1.77, 11.05) |
| 2011-2015 | IUI/OS | 73 | 1.13 (0.89, 1.42) | 1.90 (-1.69, 6.17) |
| 2016-2019 | IUI/OS | 118 | 1.28 (1.06, 1.54) | 5.76 (1.26, 10.91) |
| 2002-2005 | Clomiphene Citrate | 7 | 2.99 (1.20, 6.16) | 5.84 (0.59, 15.15) |
| 2006-2010 | Clomiphene Citrate | 28 | 1.74 (1.15, 2.51) | 3.43 (0.72, 7.04) |
| 2011-2015 | Clomiphene Citrate | 83 | 1.94 (1.55, 2.41) | 6.57 (3.81, 9.81) |
| 2016-2019 | Clomiphene Citrate | 127 | 1.74 (1.45, 2.07) | 8.14 (4.96, 11.77) |
| **Any children** | | | | |
| Nulliparous | ART | 358 | 1.57 (1.41, 1.74) | 8.79 (6.34, 11.44) |
| Parous | ART | 104 | 0.70 (0.57, 0.85) | -3.00 (-4.29, -1.52) |
| Nulliparous | IUI/OS | 211 | 1.66 (1.45, 1.90) | 10.47 (7.04, 14.26) |
| Parous | IUI/OS | 46 | 0.65 (0.47, 0.86) | -3.61 (-5.40, -1.39) |
| Nulliparous | Clomiphene Citrate | 163 | 3.36 (2.87, 3.92) | 21.32 (16.84, 26.35) |
| Parous | Clomiphene Citrate | 70 | 0.88 (0.69, 1.11) | -0.86 (-2.23, 0.79) |
| **Number of treatments** | | | | |
| 1-2 | ART | 252 | 1.20 (1.06, 1.36) | 2.37 (0.68, 4.23) |
| 3-5 | ART | 144 | 1.23 (1.04, 1.45) | 3.15 (0.51, 6.15) |
| 6+ | ART | 80 | 1.31 (1.03, 1.62) | 4.86 (0.55, 9.94) |
| 1-2 | IUI/OS | 178 | 1.34 (1.15, 1.55) | 4.48 (1.99, 7.26) |
| 3-5 | IUI/OS | 57 | 1.15 (0.87, 1.49) | 2.03 (-1.69, 6.55) |
| 6+ | IUI/OS | 37 | 1.54 (1.08, 2.12) | 8.75 (1.34, 18.23) |
| 1-2 | Clomiphene Citrate | 140 | 1.69 (1.42, 2.00) | 5.39 (3.30, 7.76) |
| 3-5 | Clomiphene Citrate | 72 | 1.92 (1.50, 2.41) | 7.25 (3.95, 11.18) |
| 6+ | Clomiphene Citrate | 33 | 2.39 (1.64, 3.35) | 11.56 (5.36, 19.60) |
| **Years since first treatment** | | | | |
| 0-1 | ART | 16 | 1.45 (0.83, 2.36) | 1.82 (-0.68, 5.45) |
| 1-2 | ART | 26 | 1.03 (0.67, 1.51) | 0.15 (-1.61, 2.51) |
| 3-5 | ART | 60 | 1.37 (1.05, 1.77) | 2.54 (0.33, 5.22) |
| 6+ | ART | 374 | 1.21 (1.09, 1.34) | 4.14 (1.81, 6.64) |
| 0-1 | IUI/OS | 9 | 1.96 (0.90, 3.73) | 3.66 (-0.39, 10.35) |
| 1-2 | IUI/OS | 12 | 1.12 (0.58, 1.95) | 0.56 (-1.97, 4.47) |
| 3-5 | IUI/OS | 24 | 1.25 (0.80, 1.86) | 1.60 (-1.28, 5.51) |
| 6+ | IUI/OS | 227 | 1.32 (1.16, 1.51) | 6.27 (3.03, 9.84) |
| 0-1 | Clomiphene Citrate | 21 | 3.81 (2.36, 5.82) | 8.82 (4.26, 15.15) |
| 1-2 | Clomiphene Citrate | 30 | 2.26 (1.52, 3.22) | 4.98 (2.07, 8.81) |
| 3-5 | Clomiphene Citrate | 44 | 1.80 (1.31, 2.42) | 4.51 (1.74, 7.98) |
| 6+ | Clomiphene Citrate | 150 | 1.65 (1.40, 1.94) | 7.80 (4.76, 11.23) |

© 2026 Vajdic CM et al. *JAMA Network Open*.

**eFigure 4. Forest Plot of SIRs and Rate Differences (Per 100 000 Person-Years) for Ovarian Cancer by MAR Cohort and Time-Varying Woman and Treatment Characteristics**

| | | Observed cancers in cohort | SIR | Rate difference |
|---|---|---|---|---|
| **Attained age group** | | | | |
| 18-35 | ART | 16 | 1.28 (0.73, 2.07) | 0.84 (-0.82, 3.25) |
| 35-39 | ART | 42 | 1.36 (0.98, 1.84) | 1.59 (-0.08, 3.68) |
| 40-44 | ART | 69 | 1.27 (0.99, 1.61) | 1.93 (-0.07, 4.33) |
| 45-49 | ART | 85 | 1.21 (0.97, 1.50) | 2.71 (-0.41, 6.37) |
| >50 | ART | 118 | 1.16 (0.96, 1.39) | 2.80 (-0.65, 6.76) |
| 18-35 | IUI/OS | 8 | 1.28 (0.55, 2.52) | 0.84 (-1.34, 4.56) |
| 35-39 | IUI/OS | 14 | 0.95 (0.52, 1.59) | -0.23 (-2.10, 2.58) |
| 40-44 | IUI/OS | 30 | 1.16 (0.78, 1.66) | 1.12 (-1.53, 4.61) |
| 45-49 | IUI/OS | 50 | 1.40 (1.04, 1.85) | 5.10 (0.53, 10.76) |
| >50 | IUI/OS | 65 | 1.10 (0.85, 1.41) | 1.84 (-2.58, 7.15) |
| 18-35 | Clomiphene Citrate | 16 | 1.26 (0.72, 2.04) | 0.79 (-0.87, 3.19) |
| 35-39 | Clomiphene Citrate | 19 | 0.95 (0.57, 1.48) | -0.22 (-1.88, 2.12) |
| 40-44 | Clomiphene Citrate | 23 | 0.88 (0.56, 1.32) | -0.83 (-3.12, 2.30) |
| 45-49 | Clomiphene Citrate | 25 | 0.97 (0.63, 1.44) | -0.32 (-4.61, 5.48) |
| >50 | Clomiphene Citrate | 22 | 1.05 (0.66, 1.59) | 0.42 (-2.75, 4.79) |
| **Calendar year** | | | | |
| 1991-2000 | ART | 12 | 1.14 (0.59, 1.99) | 0.71 (-2.07, 5.01) |
| 2001-2005 | ART | 36 | 1.75 (1.23, 2.42) | 4.88 (1.47, 9.26) |
| 2006-2010 | ART | 69 | 1.47 (1.15, 1.86) | 3.87 (1.19, 7.08) |
| 2011-2015 | ART | 99 | 1.13 (0.92, 1.37) | 1.23 (-0.80, 3.59) |
| 2016-2019 | ART | 114 | 1.10 (0.91, 1.32) | 1.04 (-0.93, 3.31) |
| 1991-2000 | IUI/OS | 9 | 1.35 (0.62, 2.57) | 1.77 (-1.91, 7.85) |
| 2001-2005 | IUI/OS | 16 | 1.27 (0.73, 2.06) | 1.74 (-1.77, 6.85) |
| 2006-2010 | IUI/OS | 40 | 1.53 (1.10, 2.09) | 4.49 (0.81, 9.15) |
| 2011-2015 | IUI/OS | 50 | 1.10 (0.82, 1.45) | 1.03 (-1.90, 4.65) |
| 2016-2019 | IUI/OS | 52 | 1.03 (0.77, 1.35) | 0.32 (-2.59, 3.90) |
| 2002-2005 | Clomiphene Citrate | 9 | 3.01 (1.37, 5.71) | 7.53 (1.41, 17.66) |
| 2006-2010 | Clomiphene Citrate | 23 | 1.43 (0.91, 2.15) | 2.00 (-0.43, 5.33) |
| 2011-2015 | Clomiphene Citrate | 33 | 0.85 (0.59, 1.20) | -0.93 (-2.61, 1.25) |
| 2016-2019 | Clomiphene Citrate | 40 | 0.84 (0.60, 1.14) | -1.15 (-2.87, 1.03) |
| **Any children** | | | | |
| Nulliparous | ART | $ | 1.54 (1.35, 1.76) | 5.61 (3.64, 7.78) |
| Parous | ART | $ | 0.80 (0.64, 0.98) | -1.53 (-2.71, -0.15) |
| Nulliparous | IUI/OS | $ | 1.48 (1.23, 1.76) | 5.04 (2.43, 8.02) |
| Parous | IUI/OS | $ | 0.75 (0.54, 1.03) | -1.89 (-3.54, 0.20) |
| Nulliparous | Clomiphene Citrate | $ | 1.67 (1.28, 2.14) | 4.64 (1.94, 7.89) |
| Parous | Clomiphene Citrate | $ | 0.63 (0.45, 0.85) | -2.16 (-3.19, -0.85) |
| **Number of treatments** | | | | |
| 1-2 | ART | 176 | 1.19 (1.02, 1.38) | 1.55 (0.15, 3.12) |
| 3-5 | ART | 93 | 1.16 (0.94, 1.42) | 1.50 (-0.60, 3.96) |
| 6+ | ART | 61 | 1.50 (1.15, 1.92) | 5.27 (1.54, 9.78) |
| 1-2 | IUI/OS | 110 | 1.20 (0.99, 1.45) | 1.84 (-0.10, 4.07) |
| 3-5 | IUI/OS | 32 | 0.94 (0.64, 1.33) | -0.56 (-3.27, 2.98) |
| 6+ | IUI/OS | 25 | 1.58 (1.02, 2.33) | 6.21 (0.24, 14.27) |
| 1-2 | Clomiphene Citrate | 63 | 0.97 (0.74, 1.24) | -0.20 (-1.58, 1.45) |
| 3-5 | Clomiphene Citrate | 28 | 0.95 (0.63, 1.37) | -0.31 (-2.29, 2.32) |
| 6+ | Clomiphene Citrate | 14 | 1.31 (0.71, 2.19) | 1.97 (-1.85, 7.69) |
| **Years since first treatment** | | | | |
| 0-1 | ART | 20 | 1.70 (1.04, 2.62) | 2.99 (0.16, 6.95) |
| 1-2 | ART | 25 | 1.01 (0.65, 1.48) | 0.03 (-1.70, 2.35) |
| 3-5 | ART | 52 | 1.34 (1.00, 1.75) | 2.04 (-0.01, 4.56) |
| 6+ | ART | 233 | 1.20 (1.05, 1.37) | 2.47 (0.65, 4.48) |
| 0-1 | IUI/OS | 12 | 2.33 (1.20, 4.06) | 5.67 (0.86, 13.09) |
| 1-2 | IUI/OS | 13 | 1.18 (0.63, 2.03) | 0.88 (-1.77, 4.91) |
| 3-5 | IUI/OS | 16 | 0.91 (0.52, 1.47) | -0.56 (-2.85, 2.78) |
| 6+ | IUI/OS | 126 | 1.17 (0.98, 1.40) | 2.09 (-0.29, 4.82) |
| 0-1 | Clomiphene Citrate | 11 | 1.72 (0.86, 3.08) | 2.63 (-0.51, 7.58) |
| 1-2 | Clomiphene Citrate | 21 | 1.53 (0.94, 2.33) | 2.16 (-0.23, 5.47) |
| 3-5 | Clomiphene Citrate | 26 | 1.18 (0.77, 1.74) | 0.93 (-1.14, 3.72) |
| 6+ | Clomiphene Citrate | 47 | 0.74 (0.55, 0.99) | -2.15 (-3.79, -0.10) |

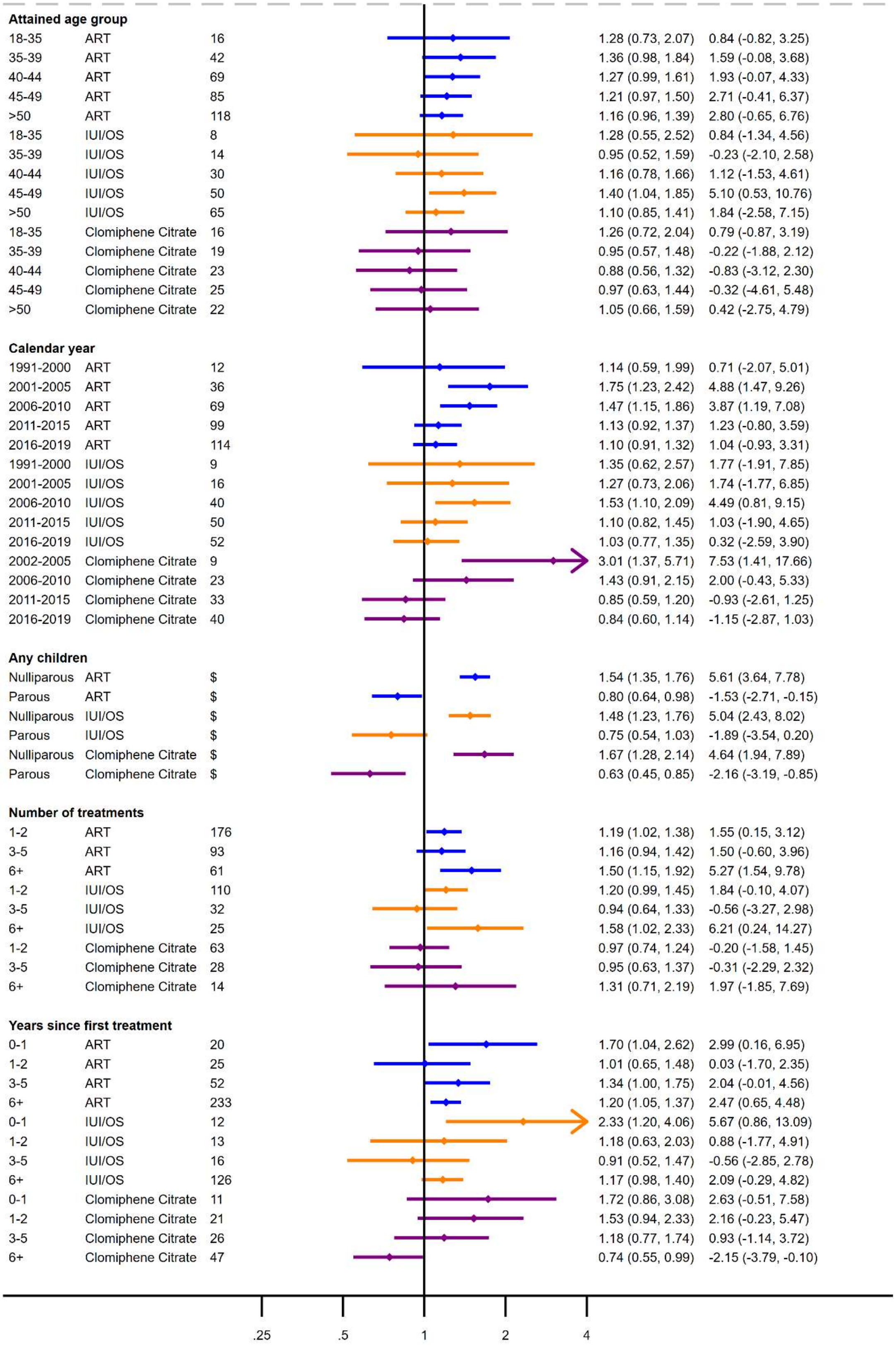


© 2026 Vajdic CM et al. *JAMA Network Open*.

Note: Number of some observed cancers masked by “$” to prevent risk of reidentification.

© 2026 Vajdic CM et al. *JAMA Network Open*.

**eFigure 5. Forest Plot of SIRs and Rate Differences (Per 100 000 Person-Years) for Invasive Melanoma by MAR Cohort and Time-Varying Woman and Treatment Characteristics**

| | | Observed cancers in cohort | SIR | Rate difference |
|---|---|---|---|---|
| **Attained age group** | | | | |
| 18-35 | ART | 118 | 1.15 (0.95, 1.37) | 3.65 (-1.27, 9.29) |
| 35-39 | ART | 278 | 1.22 (1.08, 1.37) | 7.15 (2.65, 12.07) |
| 40-44 | ART | 346 | 1.08 (0.97, 1.20) | 3.36 (-1.28, 8.39) |
| 45-49 | ART | 286 | 1.00 (0.89, 1.12) | -0.01 (-5.87, 6.39) |
| >50 | ART | 358 | 1.02 (0.91, 1.13) | 0.90 (-5.23, 7.53) |
| 18-35 | IUI/OS | 64 | 1.10 (0.85, 1.41) | 2.90 (-4.16, 11.40) |
| 35-39 | IUI/OS | 155 | 1.30 (1.10, 1.52) | 10.57 (3.63, 18.39) |
| 40-44 | IUI/OS | 192 | 1.17 (1.01, 1.35) | 7.78 (0.65, 15.71) |
| 45-49 | IUI/OS | 137 | 0.89 (0.75, 1.05) | -6.05 (-13.86, 2.81) |
| >50 | IUI/OS | 231 | 1.09 (0.96, 1.24) | 5.79 (-2.80, 15.26) |
| 18-35 | Clomiphene Citrate | 121 | 1.27 (1.06, 1.52) | 6.29 (1.32, 11.98) |
| 35-39 | Clomiphene Citrate | 167 | 1.14 (0.97, 1.32) | 4.37 (-0.97, 10.37) |
| 40-44 | Clomiphene Citrate | 184 | 1.16 (1.00, 1.34) | 6.77 (-0.20, 14.54) |
| 45-49 | Clomiphene Citrate | 123 | 1.10 (0.92, 1.32) | 5.63 (-4.48, 17.20) |
| >50 | Clomiphene Citrate | 99 | 1.09 (0.89, 1.33) | 3.18 (-3.98, 11.50) |
| **Calendar year** | | | | |
| 1991-2000 | ART | 72 | 0.95 (0.75, 1.20) | -1.64 (-9.16, 7.33) |
| 2001-2005 | ART | 136 | 1.09 (0.92, 1.29) | 3.70 (-3.23, 11.57) |
| 2006-2010 | ART | 249 | 1.12 (0.98, 1.26) | 4.50 (-0.74, 10.27) |
| 2011-2015 | ART | 421 | 1.09 (0.99, 1.20) | 3.86 (-0.43, 8.48) |
| 2016-2019 | ART | 508 | 1.06 (0.97, 1.15) | 2.68 (-1.59, 7.25) |
| 1991-2000 | IUI/OS | 52 | 1.05 (0.78, 1.37) | 1.73 (-8.17, 13.91) |
| 2001-2005 | IUI/OS | 87 | 1.09 (0.87, 1.34) | 3.65 (-5.22, 14.04) |
| 2006-2010 | IUI/OS | 130 | 1.01 (0.84, 1.19) | 0.22 (-6.66, 8.07) |
| 2011-2015 | IUI/OS | 233 | 1.13 (0.99, 1.29) | 6.10 (-0.48, 13.36) |
| 2016-2019 | IUI/OS | 277 | 1.15 (1.02, 1.29) | 7.90 (0.91, 15.55) |
| 2002-2005 | Clomiphene Citrate | 30 | 1.20 (0.81, 1.72) | 6.38 (-5.85, 22.46) |
| 2006-2010 | Clomiphene Citrate | 119 | 1.12 (0.92, 1.34) | 3.58 (-2.32, 10.34) |
| 2011-2015 | Clomiphene Citrate | 238 | 1.14 (1.00, 1.29) | 4.74 (-0.04, 10.00) |
| 2016-2019 | Clomiphene Citrate | 307 | 1.17 (1.04, 1.31) | 6.66 (1.63, 12.13) |
| **Any children** | | | | |
| Nulliparous | ART | 707 | 1.01 (0.94, 1.09) | 0.51 (-2.96, 4.17) |
| Parous | ART | 638 | 1.16 (1.07, 1.25) | 5.89 (2.62, 9.35) |
| Nulliparous | IUI/OS | 425 | 1.04 (0.94, 1.14) | 2.08 (-2.84, 7.35) |
| Parous | IUI/OS | 324 | 1.19 (1.07, 1.33) | 7.64 (2.68, 13.03) |
| Nulliparous | Clomiphene Citrate | 211 | 1.01 (0.87, 1.15) | 0.21 (-4.91, 5.88) |
| Parous | Clomiphene Citrate | 441 | 1.20 (1.09, 1.32) | 6.67 (3.08, 10.52) |
| **Number of treatments** | | | | |
| 1-2 | ART | 808 | 1.10 (1.03, 1.18) | 4.19 (1.12, 7.42) |
| 3-5 | ART | 391 | 1.04 (0.94, 1.15) | 1.77 (-2.67, 6.55) |
| 6+ | ART | 187 | 1.04 (0.89, 1.20) | 1.74 (-4.98, 9.22) |
| 1-2 | IUI/OS | 489 | 1.08 (0.99, 1.19) | 3.79 (-0.40, 8.27) |
| 3-5 | IUI/OS | 201 | 1.14 (0.99, 1.31) | 6.49 (-0.71, 14.49) |
| 6+ | IUI/OS | 89 | 1.13 (0.91, 1.39) | 6.95 (-4.91, 20.84) |
| 1-2 | Clomiphene Citrate | 402 | 1.08 (0.98, 1.19) | 2.76 (-0.85, 6.65) |
| 3-5 | Clomiphene Citrate | 223 | 1.32 (1.15, 1.50) | 11.31 (5.35, 17.90) |
| 6+ | Clomiphene Citrate | 69 | 1.13 (0.88, 1.42) | 4.64 (-4.59, 15.68) |
| **Years since first treatment** | | | | |
| <1 | ART | 89 | 1.10 (0.88, 1.35) | 2.83 (-3.56, 10.30) |
| 1-2 | ART | 181 | 1.11 (0.95, 1.28) | 3.48 (-1.49, 9.02) |
| 3-5 | ART | 270 | 1.16 (1.03, 1.31) | 5.94 (1.08, 11.26) |
| 6+ | ART | 846 | 1.04 (0.97, 1.11) | 2.06 (-1.47, 5.76) |
| <1 | IUI/OS | 44 | 1.15 (0.84, 1.55) | 4.84 (-5.12, 17.32) |
| 1-2 | IUI/OS | 100 | 1.28 (1.04, 1.56) | 9.62 (1.49, 19.05) |
| 3-5 | IUI/OS | 123 | 1.08 (0.89, 1.28) | 2.87 (-4.06, 10.81) |
| 6+ | IUI/OS | 512 | 1.08 (0.98, 1.17) | 4.11 (-0.81, 9.36) |
| <1 | Clomiphene Citrate | 38 | 0.84 (0.60, 1.16) | -4.06 (-10.39, 4.01) |
| 1-2 | Clomiphene Citrate | 112 | 1.20 (0.99, 1.44) | 5.51 (-0.39, 12.30) |
| 3-5 | Clomiphene Citrate | 167 | 1.21 (1.04, 1.41) | 6.73 (1.12, 13.03) |
| 6+ | Clomiphene Citrate | 377 | 1.15 (1.04, 1.28) | 6.62 (1.72, 11.90) |

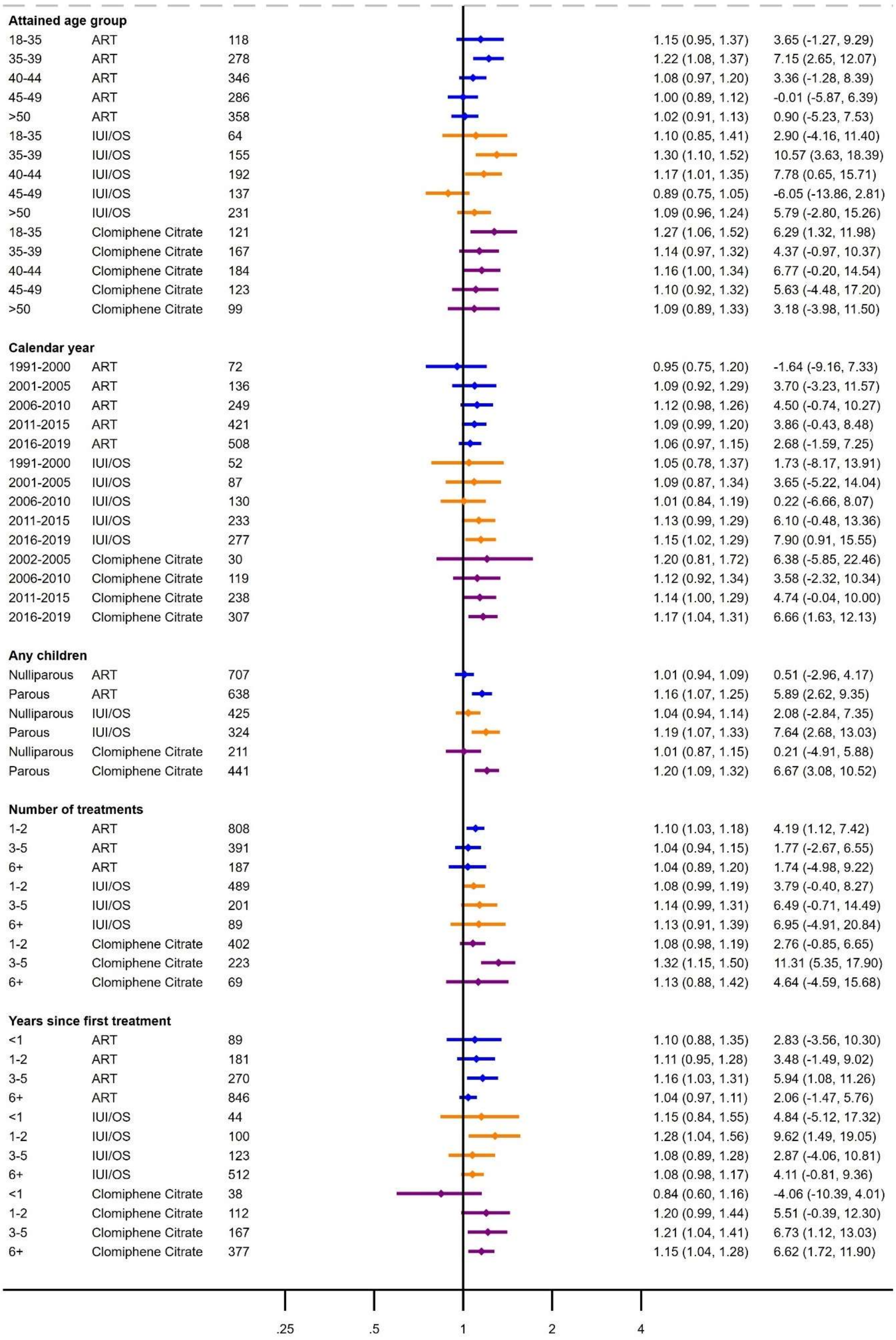


© 2026 Vajdic CM et al. *JAMA Network Open*.

**eFigure 6. Forest Plot of SIRs and Rate Differences (Per 100 000 Person-Years) for In-Situ Melanoma by MAR Cohort and Time-Varying Woman and Treatment Characteristics**

| | | Observed cancers in cohort | SIR | Rate difference |
|---|---|---|---|---|
| **Attained age group** | | | | |
| 18-35 | ART | 73 | 1.13 (0.89, 1.42) | 3.08 (-2.67, 9.91) |
| 35-39 | ART | 224 | 1.36 (1.19, 1.55) | 13.04 (6.84, 19.90) |
| 40-44 | ART | 292 | 1.17 (1.04, 1.31) | 9.43 (2.27, 17.24) |
| 45-49 | ART | 222 | 1.14 (1.00, 1.31) | 11.20 (-0.09, 23.67) |
| >50 | ART | 102 | 1.03 (0.84, 1.25) | 1.91 (-10.40, 16.17) |
| 18-35 | IUI/OS | 36 | 1.18 (0.83, 1.63) | 4.72 (-4.64, 16.73) |
| 35-39 | IUI/OS | 93 | 1.27 (1.02, 1.55) | 10.70 (0.92, 22.12) |
| 40-44 | IUI/OS | 136 | 1.27 (1.06, 1.50) | 16.05 (3.73, 30.03) |
| 45-49 | IUI/OS | 95 | 1.14 (0.92, 1.39) | 11.46 (-6.72, 32.63) |
| >50 | IUI/OS | 42 | 0.96 (0.69, 1.30) | -2.62 (-20.57, 20.00) |
| 18-35 | Clomiphene Citrate | 106 | 1.30 (1.07, 1.58) | 6.96 (1.56, 13.20) |
| 35-39 | Clomiphene Citrate | 189 | 1.35 (1.17, 1.56) | 13.17 (6.21, 20.92) |
| 40-44 | Clomiphene Citrate | 197 | 1.20 (1.04, 1.38) | 11.57 (2.16, 22.04) |
| 45-49 | Clomiphene Citrate | 124 | 1.04 (0.87, 1.24) | 3.62 (-10.78, 20.06) |
| >50 | Clomiphene Citrate | 71 | 0.86 (0.67, 1.09) | -5.74 (-13.50, 3.51) |
| **Calendar year** | | | | |
| 2011-2015 | ART | 236 | 1.07 (0.94, 1.22) | 2.76 (-2.23, 8.26) |
| 2016-2019 | ART | 598 | 1.21 (1.12, 1.32) | 14.11 (7.83, 20.77) |
| 2006-2010 | IUI/OS | 39 | 1.44 (1.02, 1.96) | 11.15 (0.53, 24.65) |
| 2011-2015 | IUI/OS | 115 | 1.15 (0.95, 1.39) | 6.51 (-1.97, 16.25) |
| 2016-2019 | IUI/OS | 248 | 1.18 (1.04, 1.33) | 12.92 (2.64, 24.22) |
| 2011-2015 | Clomiphene Citrate | 184 | 1.05 (0.91, 1.21) | 1.77 (-3.26, 7.37) |
| 2016-2019 | Clomiphene Citrate | 434 | 1.22 (1.11, 1.34) | 13.36 (6.51, 20.72) |
| **Any children** | | | | |
| Nulliparous | ART | 272 | 0.96 (0.85, 1.08) | -2.06 (-7.51, 3.91) |
| Parous | ART | 590 | 1.34 (1.24, 1.46) | 15.59 (10.77, 20.71) |
| Nulliparous | IUI/OS | 116 | 0.95 (0.78, 1.13) | -2.86 (-11.51, 7.07) |
| Parous | IUI/OS | 256 | 1.36 (1.20, 1.54) | 17.62 (9.69, 26.32) |
| Nulliparous | Clomiphene Citrate | 161 | 0.89 (0.76, 1.04) | -4.86 (-10.69, 1.70) |
| Parous | Clomiphene Citrate | 479 | 1.31 (1.20, 1.44) | 12.60 (7.98, 17.54) |
| **Number of treatments** | | | | |
| 1-2 | ART | 540 | 1.19 (1.09, 1.30) | 8.81 (4.31, 13.60) |
| 3-5 | ART | 251 | 1.15 (1.01, 1.30) | 7.61 (0.54, 15.39) |
| 6+ | ART | 122 | 1.22 (1.01, 1.46) | 12.83 (0.86, 26.52) |
| 1-2 | IUI/OS | 264 | 1.08 (0.95, 1.22) | 4.14 (-2.34, 11.25) |
| 3-5 | IUI/OS | 109 | 1.40 (1.15, 1.69) | 22.47 (8.47, 38.61) |
| 6+ | IUI/OS | 29 | 1.74 (1.16, 2.49) | 47.63 (10.51, 96.66) |
| 1-2 | Clomiphene Citrate | 415 | 1.12 (1.01, 1.23) | 4.96 (0.48, 9.78) |
| 3-5 | Clomiphene Citrate | 208 | 1.29 (1.12, 1.47) | 12.53 (5.16, 20.69) |
| 6+ | Clomiphene Citrate | 64 | 1.20 (0.93, 1.53) | 9.22 (-3.44, 24.46) |
| **Years since first treatment** | | | | |
| <1 | ART | 63 | 1.04 (0.80, 1.33) | 1.14 (-5.87, 9.61) |
| 1-2 | ART | 141 | 1.09 (0.91, 1.28) | 2.93 (-2.97, 9.61) |
| 3-5 | ART | 221 | 1.14 (1.00, 1.31) | 6.29 (-0.07, 13.32) |
| 6+ | ART | 488 | 1.26 (1.15, 1.38) | 17.95 (10.39, 26.04) |
| <1 | IUI/OS | 15 | 0.60 (0.34, 1.00) | -12.31 (-20.60, -0.08) |
| 1-2 | IUI/OS | 56 | 1.03 (0.78, 1.34) | 1.26 (-8.05, 12.64) |
| 3-5 | IUI/OS | 105 | 1.27 (1.04, 1.54) | 12.75 (1.90, 25.29) |
| 6+ | IUI/OS | 226 | 1.28 (1.11, 1.45) | 20.45 (8.50, 33.64) |
| <1 | Clomiphene Citrate | 30 | 0.87 (0.58, 1.24) | -2.98 (-9.28, 5.30) |
| 1-2 | Clomiphene Citrate | 90 | 1.12 (0.90, 1.38) | 3.39 (-2.61, 10.41) |
| 3-5 | Clomiphene Citrate | 158 | 1.17 (0.99, 1.36) | 6.10 (-0.26, 13.25) |
| 6+ | Clomiphene Citrate | 409 | 1.21 (1.10, 1.34) | 13.49 (6.26, 21.26) |

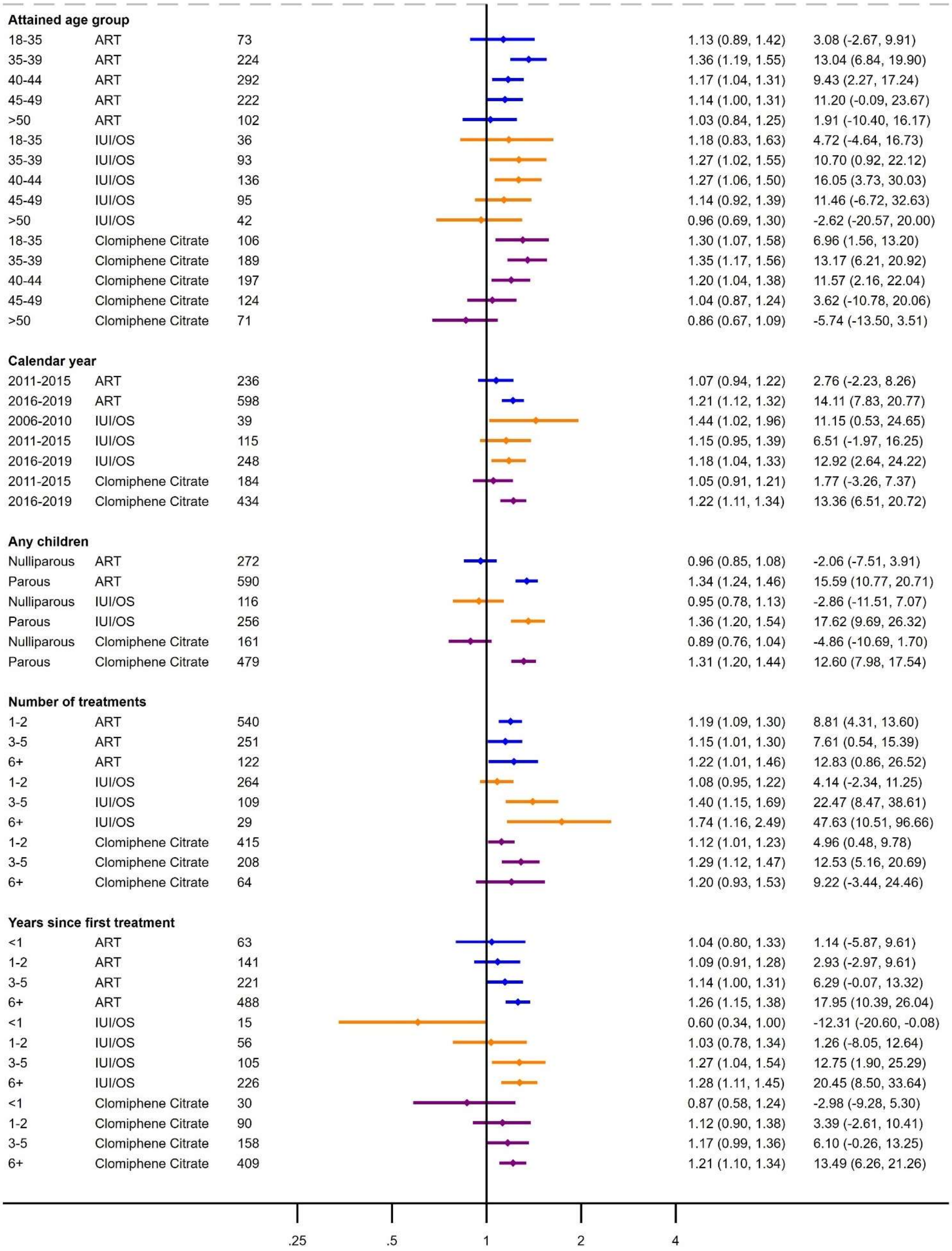


Note: Some categories had small numbers of observed cases and could not be reported.

© 2026 Vajdic CM et al. *JAMA Network Open*.

## eFigure 7. Forest Plot of SIRs and Rate Differences (Per 100 000 Person-Years) for Colorectal Cancer by MAR Cohort and Time-Varying Woman and Treatment Characteristics

| | | Observed cancers in cohort | SIR | Rate difference |
|---|---|---|---|---|
| **Attained age group** | | | | |
| 18-35 | ART | 35 | 1.13 (0.79, 1.57) | 0.96 (-1.61, 4.26) |
| 35-39 | ART | 100 | 1.23 (1.00, 1.49) | 2.62 (-0.03, 5.69) |
| 40-44 | ART | 144 | 1.00 (0.85, 1.18) | 0.08 (-2.88, 3.42) |
| 45-49 | ART | 158 | 0.91 (0.78, 1.07) | -2.79 (-7.10, 2.06) |
| >50 | ART | 276 | 0.93 (0.82, 1.05) | -3.56 (-8.92, 2.31) |
| 18-35 | IUI/OS | 15 | 1.00 (0.56, 1.64) | -0.03 (-3.20, 4.64) |
| 35-39 | IUI/OS | 47 | 1.24 (0.91, 1.64) | 2.65 (-1.04, 7.23) |
| 40-44 | IUI/OS | 73 | 1.07 (0.84, 1.35) | 1.38 (-2.91, 6.49) |
| 45-49 | IUI/OS | 72 | 0.81 (0.64, 1.02) | -5.87 (-11.43, 0.76) |
| >50 | IUI/OS | 143 | 0.83 (0.70, 0.98) | -8.85 (-15.55, -1.27) |
| 18-35 | Clomiphene Citrate | 40 | 1.19 (0.85, 1.62) | 1.54 (-1.22, 5.03) |
| 35-39 | Clomiphene Citrate | 61 | 1.06 (0.81, 1.37) | 0.81 (-2.33, 4.62) |
| 40-44 | Clomiphene Citrate | 84 | 1.15 (0.92, 1.42) | 2.97 (-1.65, 8.40) |
| 45-49 | Clomiphene Citrate | 49 | 0.75 (0.55, 0.99) | -8.06 (-14.27, -0.38) |
| >50 | Clomiphene Citrate | 54 | 0.90 (0.68, 1.18) | -2.24 (-7.43, 4.13) |
| **Calendar year** | | | | |
| 1991-2000 | ART | 20 | 0.98 (0.60, 1.52) | -0.17 (-3.91, 5.06) |
| 2001-2005 | ART | 49 | 1.07 (0.79, 1.42) | 1.07 (-2.96, 6.07) |
| 2006-2010 | ART | 110 | 0.99 (0.81, 1.19) | -0.19 (-3.62, 3.76) |
| 2011-2015 | ART | 217 | 0.95 (0.83, 1.08) | -1.27 (-4.32, 2.11) |
| 2016-2019 | ART | 317 | 0.99 (0.88, 1.10) | -0.36 (-3.72, 3.29) |
| 1991-2000 | IUI/OS | 12 | 0.94 (0.48, 1.64) | -0.61 (-4.98, 6.12) |
| 2001-2005 | IUI/OS | 26 | 0.92 (0.60, 1.35) | -1.12 (-5.73, 5.08) |
| 2006-2010 | IUI/OS | 62 | 0.97 (0.74, 1.24) | -0.61 (-5.27, 5.02) |
| 2011-2015 | IUI/OS | 113 | 0.95 (0.78, 1.14) | -1.46 (-5.97, 3.74) |
| 2016-2019 | IUI/OS | 137 | 0.87 (0.73, 1.03) | -4.64 (-9.49, 0.88) |
| 2002-2005 | Clomiphene Citrate | 6 | 1.03 (0.38, 2.25) | 0.24 (-4.53, 9.08) |
| 2006-2010 | Clomiphene Citrate | 35 | 0.97 (0.68, 1.35) | -0.30 (-3.37, 3.65) |
| 2011-2015 | Clomiphene Citrate | 112 | 1.16 (0.95, 1.39) | 2.51 (-0.72, 6.23) |
| 2016-2019 | Clomiphene Citrate | 135 | 0.89 (0.75, 1.06) | -2.40 (-5.68, 1.34) |
| **Any children** | | | | |
| Nulliparous | ART | 389 | 0.95 (0.86, 1.05) | -1.24 (-3.80, 1.51) |
| Parous | ART | 306 | 1.02 (0.91, 1.14) | 0.40 (-1.84, 2.84) |
| Nulliparous | IUI/OS | 198 | 0.87 (0.75, 1.00) | -3.67 (-6.98, 0.02) |
| Parous | IUI/OS | 136 | 0.96 (0.81, 1.14) | -0.82 (-3.98, 2.78) |
| Nulliparous | Clomiphene Citrate | 103 | 1.04 (0.85, 1.26) | 0.72 (-2.80, 4.80) |
| Parous | Clomiphene Citrate | 174 | 0.97 (0.84, 1.13) | -0.41 (-2.63, 2.08) |
| **Number of treatments** | | | | |
| 1-2 | ART | 388 | 0.97 (0.88, 1.07) | -0.67 (-2.78, 1.60) |
| 3-5 | ART | 216 | 1.00 (0.87, 1.14) | -0.01 (-3.28, 3.60) |
| 6+ | ART | 109 | 0.99 (0.81, 1.19) | -0.31 (-5.38, 5.53) |
| 1-2 | IUI/OS | 225 | 0.91 (0.79, 1.04) | -2.22 (-5.04, 0.88) |
| 3-5 | IUI/OS | 79 | 0.86 (0.68, 1.07) | -3.51 (-7.92, 1.72) |
| 6+ | IUI/OS | 46 | 1.08 (0.79, 1.44) | 2.20 (-6.14, 12.60) |
| 1-2 | Clomiphene Citrate | 174 | 0.97 (0.83, 1.13) | -0.46 (-2.80, 2.16) |
| 3-5 | Clomiphene Citrate | 85 | 1.05 (0.84, 1.30) | 0.84 (-2.76, 5.08) |
| 6+ | Clomiphene Citrate | 29 | 0.98 (0.66, 1.41) | -0.29 (-6.06, 7.33) |
| **Years since first treatment** | | | | |
| <1 | ART | 30 | 1.05 (0.71, 1.49) | 0.48 (-3.07, 5.15) |
| 1-2 | ART | 68 | 1.08 (0.84, 1.37) | 1.04 (-1.93, 4.59) |
| 3-5 | ART | 103 | 1.03 (0.84, 1.24) | 0.41 (-2.53, 3.82) |
| 6+ | ART | 512 | 0.96 (0.88, 1.04) | -1.42 (-4.15, 1.49) |
| <1 | IUI/OS | 13 | 1.07 (0.57, 1.83) | 0.72 (-4.31, 8.37) |
| 1-2 | IUI/OS | 26 | 0.96 (0.63, 1.41) | -0.44 (-4.37, 4.84) |
| 3-5 | IUI/OS | 44 | 0.98 (0.71, 1.32) | -0.28 (-4.29, 4.75) |
| 6+ | IUI/OS | 267 | 0.89 (0.79, 1.01) | -3.55 (-7.08, 0.31) |
| <1 | Clomiphene Citrate | 16 | 1.04 (0.60, 1.69) | 0.38 (-3.53, 6.07) |
| 1-2 | Clomiphene Citrate | 44 | 1.26 (0.92, 1.69) | 2.72 (-0.87, 7.22) |
| 3-5 | Clomiphene Citrate | 60 | 1.02 (0.78, 1.31) | 0.24 (-3.03, 4.21) |
| 6+ | Clomiphene Citrate | 168 | 0.93 (0.80, 1.08) | -1.61 (-4.84, 2.01) |

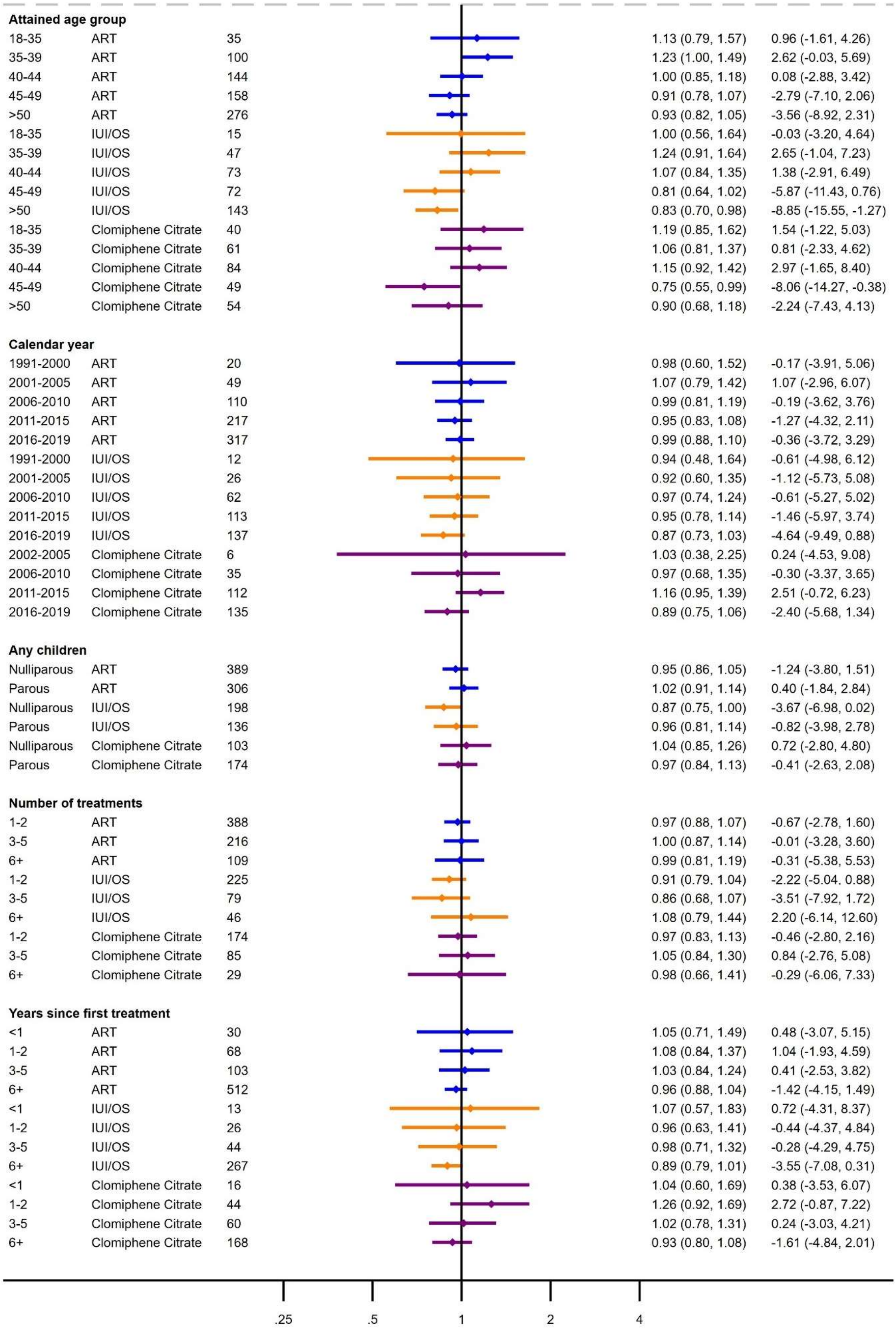


© 2026 Vajdic CM et al. *JAMA Network Open*.

**eFigure 8. Forest Plot of SIRs and Rate Differences (Per 100 000 Person-Years) for Thyroid Cancer by MAR Cohort and Time-Varying Woman and Treatment Characteristics**

| | | Observed cancers in cohort | SIR | Rate difference |
|---|---|---|---|---|
| **Attained age group** | | | | |
| 18-35 | ART | 86 | 1.31 (1.05, 1.62) | 4.91 (0.75, 9.80) |
| 35-39 | ART | 127 | 0.91 (0.76, 1.08) | -1.83 (-4.83, 1.59) |
| 40-44 | ART | 184 | 1.02 (0.88, 1.18) | 0.41 (-2.94, 4.15) |
| 45-49 | ART | 157 | 1.04 (0.88, 1.21) | 0.99 (-3.31, 5.83) |
| >50 | ART | 182 | 1.11 (0.95, 1.28) | 3.03 (-1.29, 7.86) |
| 18-35 | IUI/OS | 36 | 1.13 (0.79, 1.56) | 1.96 (-3.21, 8.60) |
| 35-39 | IUI/OS | 60 | 0.91 (0.70, 1.17) | -1.73 (-5.94, 3.37) |
| 40-44 | IUI/OS | 95 | 1.11 (0.90, 1.35) | 2.51 (-2.43, 8.26) |
| 45-49 | IUI/OS | 73 | 0.94 (0.74, 1.18) | -1.60 (-7.21, 5.07) |
| >50 | IUI/OS | 97 | 1.04 (0.85, 1.27) | 1.22 (-4.24, 7.57) |
| 18-35 | Clomiphene Citrate | 82 | 1.17 (0.93, 1.45) | 2.83 (-1.22, 7.60) |
| 35-39 | Clomiphene Citrate | 115 | 1.16 (0.96, 1.39) | 3.45 (-0.95, 8.51) |
| 40-44 | Clomiphene Citrate | 125 | 1.32 (1.10, 1.57) | 8.24 (2.55, 14.75) |
| 45-49 | Clomiphene Citrate | 74 | 1.22 (0.95, 1.53) | 6.39 (-1.35, 15.58) |
| >50 | Clomiphene Citrate | 50 | 1.01 (0.75, 1.33) | 0.16 (-4.82, 6.31) |
| **Calendar year** | | | | |
| 1991-2000 | ART | 32 | 1.42 (0.97, 2.00) | 4.53 (-0.32, 10.86) |
| 2001-2005 | ART | 51 | 1.00 (0.75, 1.32) | 0.08 (-4.04, 5.16) |
| 2006-2010 | ART | 102 | 0.88 (0.72, 1.07) | -2.41 (-5.71, 1.41) |
| 2011-2015 | ART | 241 | 1.04 (0.91, 1.18) | 1.03 (-2.19, 4.58) |
| 2016-2019 | ART | 310 | 1.10 (0.98, 1.23) | 2.82 (-0.50, 6.43) |
| 1991-2000 | IUI/OS | 17 | 1.18 (0.69, 1.89) | 1.97 (-3.37, 9.65) |
| 2001-2005 | IUI/OS | 20 | 0.64 (0.39, 0.98) | -5.83 (-9.81, -0.26) |
| 2006-2010 | IUI/OS | 57 | 0.88 (0.67, 1.15) | -2.40 (-6.85, 3.03) |
| 2011-2015 | IUI/OS | 130 | 1.14 (0.95, 1.36) | 3.67 (-1.19, 9.21) |
| 2016-2019 | IUI/OS | 137 | 1.05 (0.89, 1.25) | 1.57 (-3.29, 7.09) |
| 2002-2005 | Clomiphene Citrate | 8 | 0.73 (0.32, 1.44) | -3.70 (-9.39, 6.04) |
| 2006-2010 | Clomiphene Citrate | 74 | 1.23 (0.97, 1.55) | 4.03 (-0.56, 9.50) |
| 2011-2015 | Clomiphene Citrate | 161 | 1.19 (1.02, 1.39) | 4.24 (0.34, 8.63) |
| 2016-2019 | Clomiphene Citrate | 203 | 1.20 (1.04, 1.38) | 5.17 (1.11, 9.68) |
| **Any children** | | | | |
| Nulliparous | ART | 349 | 1.03 (0.93, 1.14) | 0.70 (-1.72, 3.31) |
| Parous | ART | 366 | 1.06 (0.95, 1.17) | 1.34 (-1.12, 4.00) |
| Nulliparous | IUI/OS | 170 | 0.93 (0.80, 1.08) | -1.59 (-4.66, 1.84) |
| Parous | IUI/OS | 172 | 1.08 (0.92, 1.25) | 1.77 (-1.81, 5.77) |
| Nulliparous | Clomiphene Citrate | 113 | 0.97 (0.80, 1.17) | -0.64 (-4.34, 3.62) |
| Parous | Clomiphene Citrate | 316 | 1.29 (1.15, 1.44) | 6.27 (3.25, 9.56) |
| **Number of treatments** | | | | |
| 1-2 | ART | 444 | 1.10 (1.00, 1.21) | 2.23 (-0.03, 4.66) |
| 3-5 | ART | 190 | 0.94 (0.81, 1.08) | -1.43 (-4.49, 1.97) |
| 6+ | ART | 102 | 1.07 (0.87, 1.29) | 1.65 (-3.25, 7.32) |
| 1-2 | IUI/OS | 228 | 0.98 (0.86, 1.12) | -0.47 (-3.30, 2.66) |
| 3-5 | IUI/OS | 85 | 0.99 (0.79, 1.22) | -0.32 (-4.91, 5.08) |
| 6+ | IUI/OS | 48 | 1.37 (1.01, 1.82) | 8.77 (0.23, 19.36) |
| 1-2 | Clomiphene Citrate | 268 | 1.16 (1.02, 1.30) | 3.39 (0.46, 6.60) |
| 3-5 | Clomiphene Citrate | 114 | 1.09 (0.90, 1.30) | 1.90 (-2.30, 6.74) |
| 6+ | Clomiphene Citrate | 64 | 1.70 (1.31, 2.17) | 15.86 (6.99, 26.54) |
| **Years since first treatment** | | | | |
| <1 | ART | 45 | 0.95 (0.69, 1.27) | -0.88 (-5.32, 4.66) |
| 1-2 | ART | 96 | 1.00 (0.81, 1.22) | 0.00 (-3.56, 4.15) |
| 3-5 | ART | 144 | 1.08 (0.91, 1.27) | 1.56 (-1.95, 5.54) |
| 6+ | ART | 451 | 1.06 (0.97, 1.16) | 1.65 (-0.91, 4.39) |
| <1 | IUI/OS | 14 | 0.71 (0.39, 1.19) | -4.69 (-9.95, 3.17) |
| 1-2 | IUI/OS | 45 | 1.10 (0.81, 1.48) | 1.85 (-3.47, 8.49) |
| 3-5 | IUI/OS | 61 | 1.02 (0.78, 1.32) | 0.47 (-4.32, 6.27) |
| 6+ | IUI/OS | 241 | 1.03 (0.90, 1.17) | 0.80 (-2.54, 4.48) |
| <1 | Clomiphene Citrate | 19 | 0.69 (0.42, 1.08) | -4.84 (-9.15, 1.24) |
| 1-2 | Clomiphene Citrate | 70 | 1.19 (0.93, 1.50) | 3.34 (-1.27, 8.84) |
| 3-5 | Clomiphene Citrate | 110 | 1.23 (1.01, 1.49) | 4.80 (0.29, 10.00) |
| 6+ | Clomiphene Citrate | 247 | 1.24 (1.09, 1.40) | 6.31 (2.37, 10.64) |

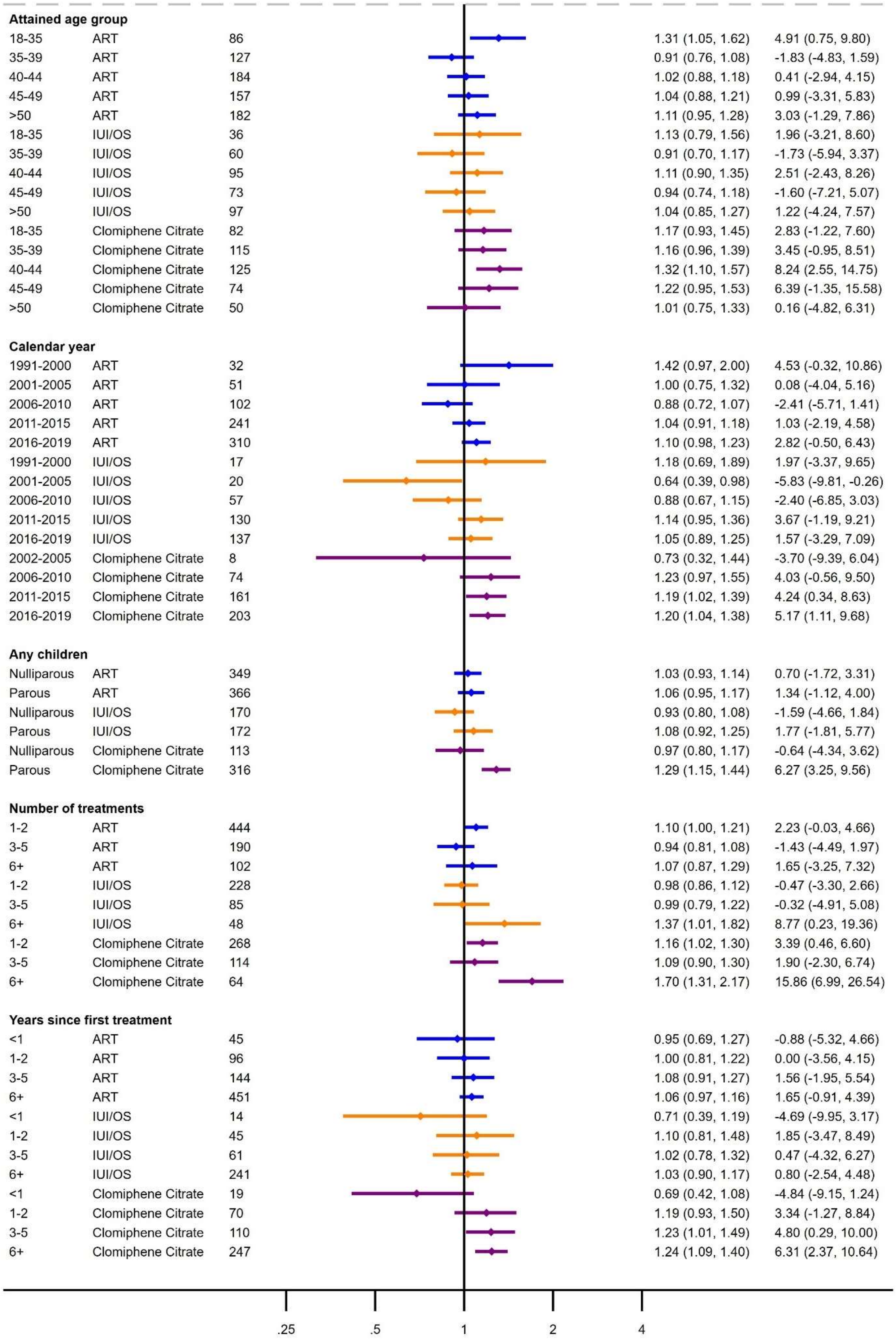


© 2026 Vajdic CM et al. *JAMA Network Open*.

## eFigure 9. Forest Plot of SIRs and Rate Difference (Per 100 000 Person-Years) for Uterine Cancer Morphology Subtypes by MAR Cohort

| | Observed cancers in cohort | SIR | Rate difference |
|---|---|---|---|
| **All uterine** | | | |
| ART | 476 | 1.23 (1.12, 1.34) | 2.91 (1.53, 4.39) |
| IUI/OS | 272 | 1.32 (1.17, 1.49) | 4.29 (2.25, 6.54) |
| Clomiphene Citrate | 245 | 1.83 (1.60, 2.07) | 6.51 (4.76, 8.43) |
| **Endometrioid adenocarcinoma** | | | |
| ART | 356 | 1.20 (1.08, 1.33) | 1.95 (0.76, 3.24) |
| IUI/OS | 209 | 1.32 (1.15, 1.51) | 3.32 (1.53, 5.30) |
| Clomiphene Citrate | 206 | 1.97 (1.71, 2.26) | 5.97 (4.37, 7.74) |
| **Adenocarcinoma NOS** | | | |
| ART | 21 | 1.38 (0.85, 2.11) | 0.19 (-0.07, 0.56) |
| IUI/OS | 13 | 1.55 (0.83, 2.66) | 0.30 (-0.09, 0.91) |
| **Serous/Papillary serous** | | | |
| ART | 8 | 0.83 (0.36, 1.64) | -0.05 (-0.20, 0.20) |
| IUI/OS | 8 | 1.50 (0.65, 2.95) | 0.17 (-0.12, 0.68) |
| **Sarcoma (excluding adenocarcinoma)** | | | |
| ART | 54 | 1.35 (1.02, 1.77) | 0.47 (0.02, 1.01) |
| IUI/OS | 24 | 1.20 (0.77, 1.78) | 0.26 (-0.31, 1.02) |
| Clomiphene Citrate | 22 | 1.35 (0.85, 2.05) | 0.34 (-0.15, 1.00) |
| **Type 1** | | | |
| ART | 379 | 1.21 (1.09, 1.34) | 2.16 (0.93, 3.49) |
| IUI/OS | 223 | 1.33 (1.16, 1.52) | 3.63 (1.78, 5.67) |
| Clomiphene Citrate | 210 | 1.94 (1.69, 2.22) | 5.99 (4.37, 7.77) |
| **Type 2** | | | |
| ART | 12 | 0.95 (0.49, 1.67) | -0.02 (-0.21, 0.28) |
| IUI/OS | 10 | 1.46 (0.70, 2.69) | 0.21 (-0.13, 0.76) |

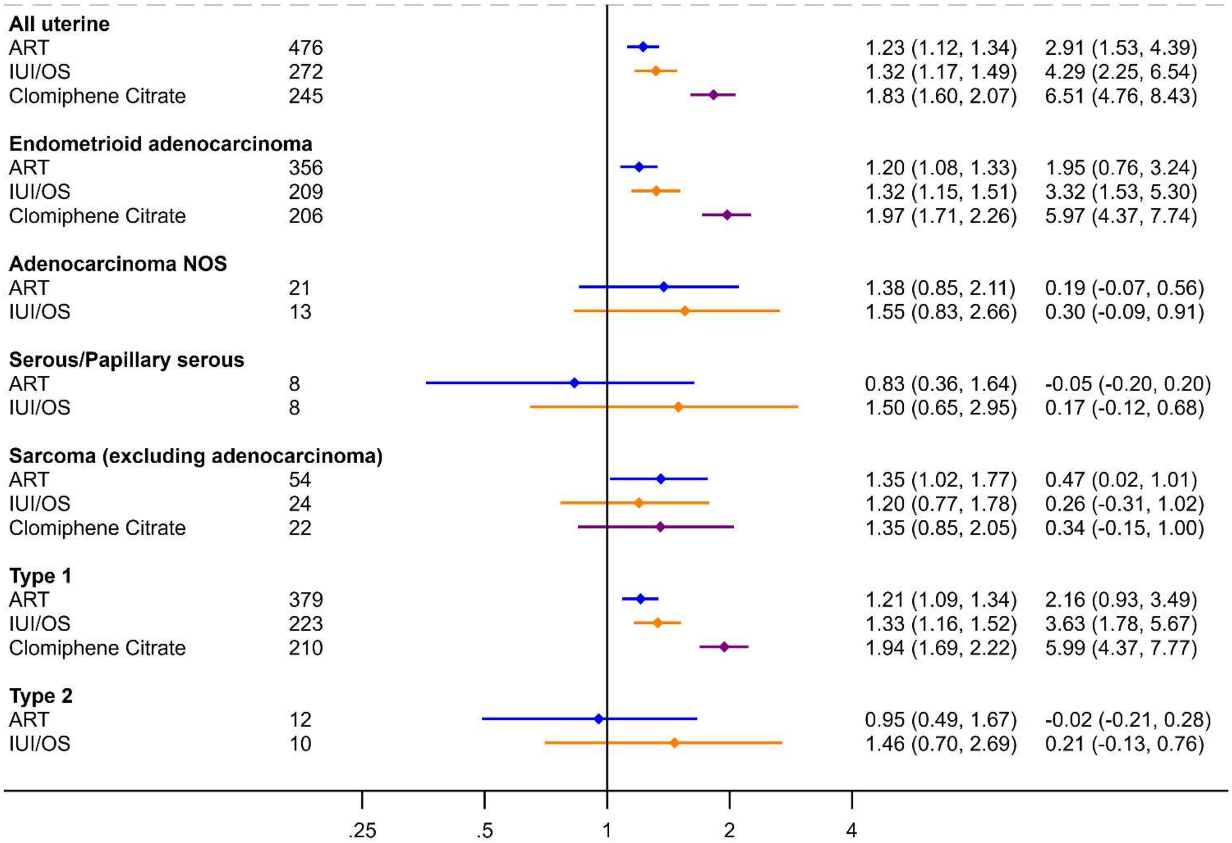


© 2026 Vajdic CM et al. *JAMA Network Open*.

**eFigure 10. Forest Plot of SIRs and Rate Difference (Per 100 000 Person-Years) for Ovarian Cancer Morphology Subtypes by MAR Cohort**
**FGO: Female genital organs**

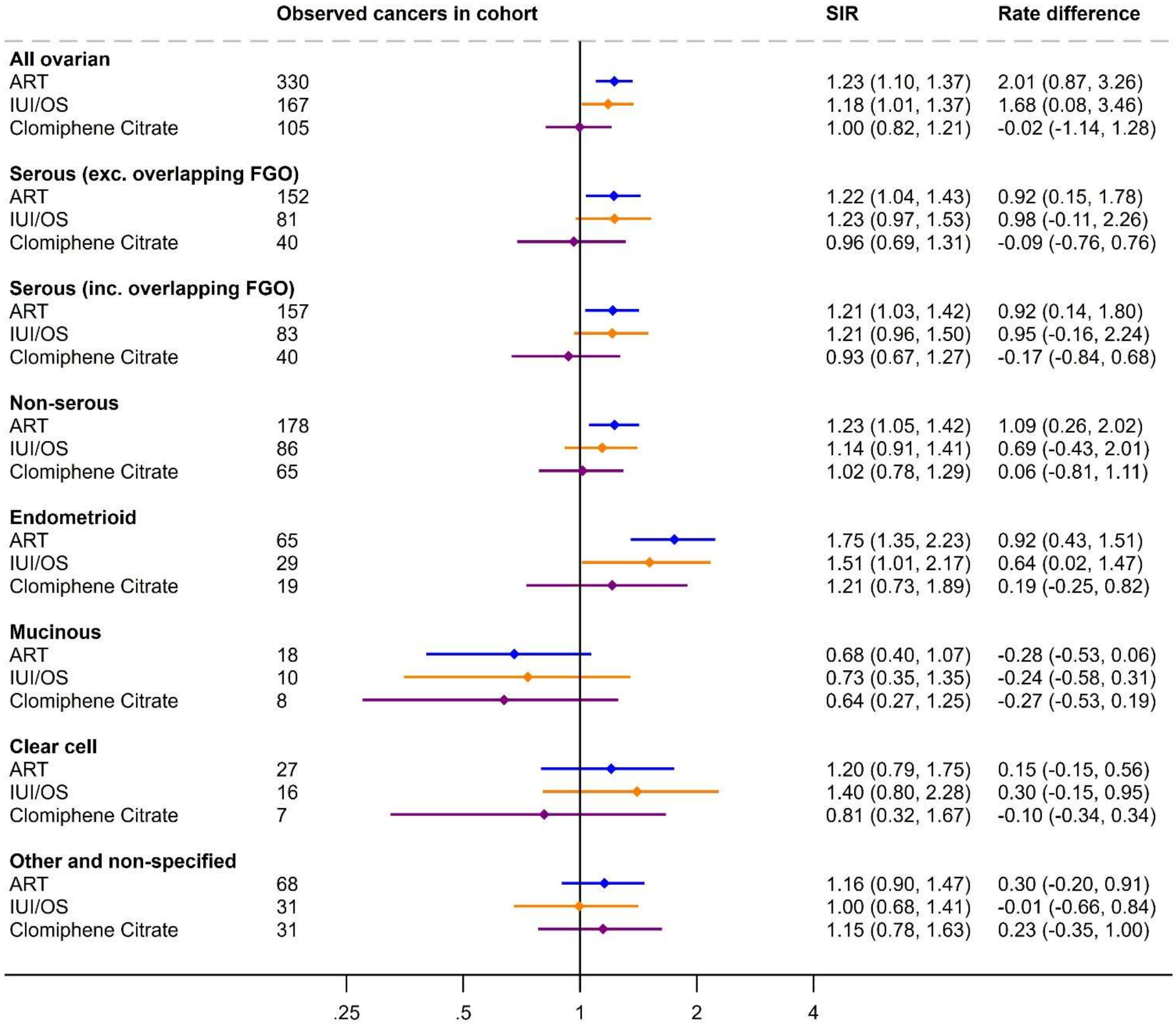


© 2026 Vajdic CM et al. *JAMA Network Open*.

**eFigure 11. Forest Plot of SIRs and Rate Difference (Per 100 000 Person-Years) for Hematological Cancer Subtypes by MAR Cohort**

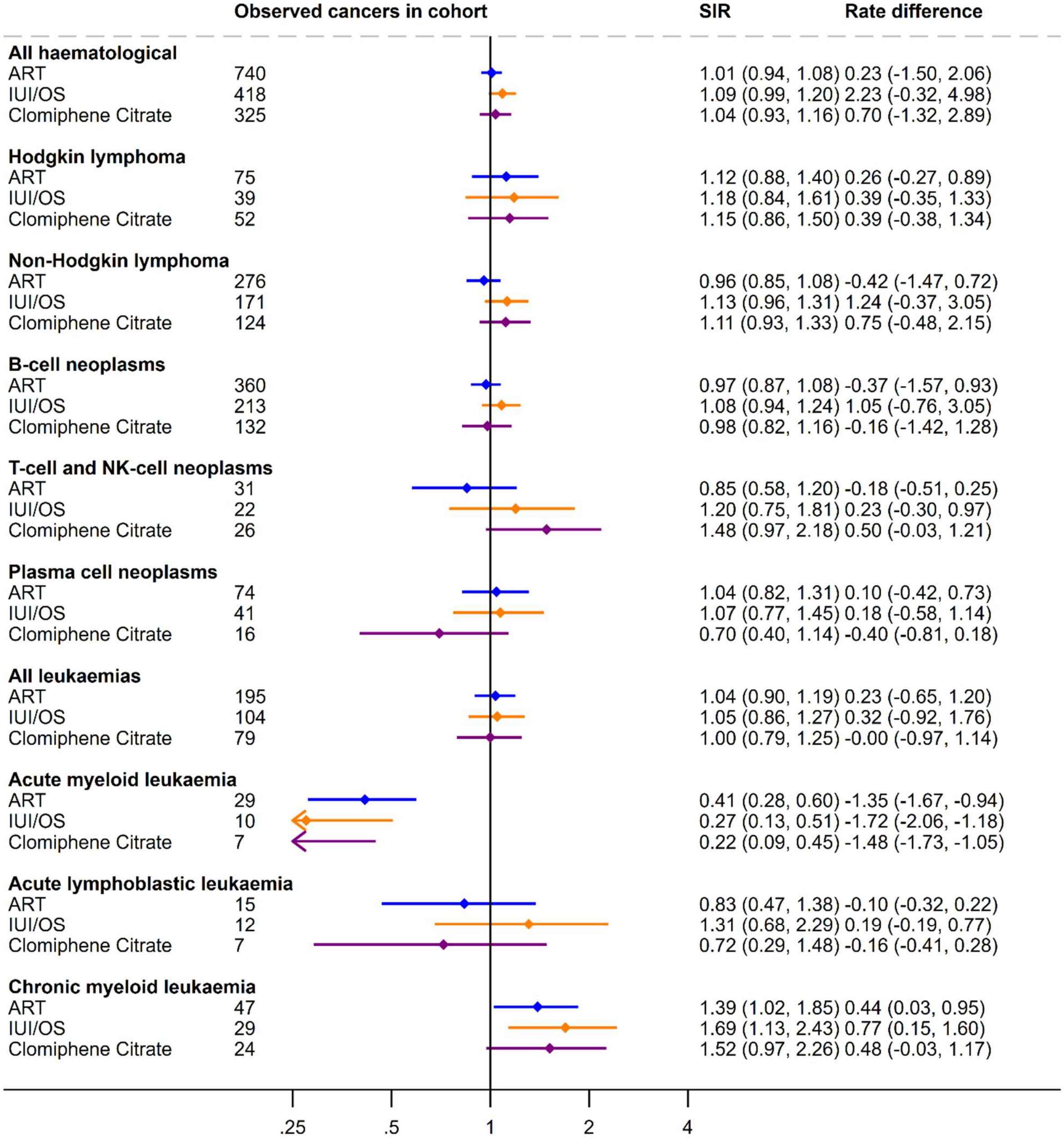


© 2026 Vajdic CM et al. *JAMA Network Open*.
