# Supplementary material for: Cancer Incidence in Women After Medically Assisted Reproduction
Source: JAMA Netw Open. 2026 Mar 10;9(3):e261332. doi: 10.1001/jamanetworkopen.2026.1332 (PMC12976796; doi:10.1001/jamanetworkopen.2026.1332)
Supplement: Supplement 2. — Data Sharing Statement [file jamanetwopen-e261332-s002.pdf]

Data Sharing Statement

Vajdic. Cancer Incidence in Women After Medically Assisted Reproduction. *JAMA Netw Open*. Published March 10, 2026. doi:10.1001/jamanetworkopen.2026.1332

Data
Data available: No

Additional Information
Explanation for why data not available: In keeping with Australian privacy and confidentiality legislation, the individual person data accessed and used for this project are not publicly available and cannot be shared by the authors. Data access can be requested from the data custodians and the Australian Institute of Health and Welfare (https://www.aihw.gov.au/our-services/data-linkage). All analysis code is available.
